# GSK3β-Dzip1-Rab8 Cascade Regulates Ciliogenesis after Mitosis

**DOI:** 10.1371/journal.pbio.1002129

**Published:** 2015-04-10

**Authors:** Boyan Zhang, Tingting Zhang, Guopeng Wang, Gang Wang, Wangfei Chi, Qing Jiang, Chuanmao Zhang

**Affiliations:** The Key Laboratory of Cell Proliferation and Differentiation of the Ministry of Education and the State Key Laboratory of Biomembrane and Membrane Biotechnology, College of Life Sciences, Peking University, Beijing, China; Institut Curie, UNITED STATES

## Abstract

The primary cilium, which disassembles before mitotic entry and reassembles after mitosis, organizes many signal transduction pathways that are crucial for cell life and individual development. However, how ciliogenesis is regulated during the cell cycle remains largely unknown. Here we show that GSK3β, Dzip1, and Rab8 co-regulate ciliogenesis by promoting the assembly of the ciliary membrane after mitosis. Immunofluorescence and super-resolution microscopy showed that Dzip1 was localized to the periciliary diffusion barrier and enriched at the mother centriole. Knockdown of Dzip1 by short hairpin RNAs led to failed ciliary localization of Rab8, and Rab8 accumulation at the basal body. Dzip1 preferentially bound to Rab8^GDP^ and promoted its dissociation from its inhibitor GDI2 at the pericentriolar region, as demonstrated by sucrose gradient centrifugation of purified basal bodies, immunoprecipitation, and acceptor-bleaching fluorescence resonance energy transfer assays. By means of in vitro phosphorylation, in vivo gel shift, phospho-peptide identification by mass spectrometry, and GST pulldown assays, we demonstrated that Dzip1 was phosphorylated by GSK3β at S520 in G0 phase, which increased its binding to GDI2 to promote the release of Rab8^GDP^ at the cilium base. Moreover, ciliogenesis was inhibited by overexpression of the GSK3β-nonphosphorylatable Dzip1 mutant or by disabling of GSK3β by specific inhibitors or knockout of GSK3β in cells. Collectively, our data reveal a unique cascade consisting of GSK3β, Dzip1, and Rab8 that regulates ciliogenesis after mitosis.

## Abstract 2

The asymmetric assembly of a new primary cilium in one of two daughter cells after mitosis is regulated by a cascade involving GSK3β, Dzip1, and Rab8.

## Author Summary

The primary cilium is an antenna-like organelle that projects out from the surface of cells and is present in almost all vertebrate cells, playing crucial roles in many cellular processes, including chemical sensation, signal transduction, and control of cell growth. The primary cilium assembles via a dynamic process called ciliogenesis that is regulated during the cell cycle: it assembles after mitosis and disassembles again before entering the next mitotic cycle. Here we investigate the regulatory mechanisms underlying this process. We show that Dzip1—a protein known to promote ciliogenesis—is preferentially recruited to the centrosome of the daughter cell that contains the grandmother centriole. Once in the centrosome, Dzip1 promotes release of Rab8^GDP^—a small GTPase that regulates membrane vesicular trafficking to the cilium—from its inhibitor GDI2 at the pericentriolar region, thereby facilitating ciliogenesis. This process is further regulated by an enzyme, GSK3β, whose increased kinase activity during the M- to G0-phase transition of the cell cycle results in phosphorylation of Dzip1, promoting the ability of Dzip1 to release Rab8^GDP^. Our findings identify the molecular mechanism underlying the GSK3β-Dzip1-Rab8 signaling cascade, shedding light on how ciliogenesis is coordinated with mitotic exit. They also provide an understanding of why ciliogenesis always takes place earlier in one of the two daughter cells.

## Introduction

The primary cilium is an antenna-like organelle projecting from the apical surface of most vertebrate cells and plays pivotal roles in mediating signal transduction for the cell and regulating the balance between cell proliferation and differentiation [1–4]. It consists of a basal body, a microtubule-based axoneme generated from the basal body, and a signaling-receptor-enriched ciliary membrane sheet extending from the cell membrane. Between the ciliary membrane sheet and the cell membrane, there is a periciliary diffusion barrier (PDB), a transition zone that forms a selective barrier to the membrane proteins that are laterally transported on the membranes [5,6]. The primary cilium is also gated by the pinwheel-shaped transition fibers that originate from the distal appendages of the basal body and end at the “cilium necklace.” Bidirectional transport of ciliary proteins between the cytoplasm and the cilium is mediated by a multiprotein complex, the IFT (intraflagellar transport) machinery [7].

The primary cilium is structurally dynamic during the cell cycle. It disassembles before the mitotic entry and reassembles at the end of mitosis [8,9]. Building a cilium, or ciliogenesis, is a sequentially coordinated process [10,11], during which polarized membrane vesicle trafficking to, and fusion with, the cell membrane—mediated by vesicle-bound Rab GTPases—is of great importance for formation of the ciliary membrane sheet [10,12]. Among the Rab GTPases, Rab8 is a core modulator of membrane vesicle trafficking to cilium, and specifically functions at the steps of vesicle docking and fusion with the cell membrane [13]. Rab8 in its GTP-bound form (Rab8^GTP^) is active and can be converted into the inactive form (Rab8^GDP^) by hydrolysis of the GTP molecule, which is mediated by its GTPase-activating protein. Conversely, the conversion of Rab8^GDP^ to Rab8^GTP^ requires several specific factors including GDP-dissociating inhibitor protein (GDI), GDI displacement factor (GDF), and Rab8’s guanine nucleotide exchange factor (GEF), Rabin8 [14–16]. The GTP/GDP-bound status of Rab8 has antagonistic effects on ciliogenesis: overexpression of the Rab8^GDP^-mimicking mutant Rab8^T22N^ blocks cilium assembly, whereas overexpression of the Rab8^GTP^-mimicking mutant Rab8^Q67L^ promotes cilium assembly [17]. Both the proper localization and the efficient GTP-GDP cycling of vesicle-bound Rabs are important for vesicle trafficking and ciliogenesis [15].

Dzip1 is a zinc-finger-containing protein that is predominantly expressed in human embryonic stem cells and germ cells [18]. The *Dzip1* gene was first identified in zebrafish (where it is called *iguana*), and its mutation results in failure of ciliogenesis in Kupffer’s vesicle cells [19,20]. In cultured mammalian cells, Dzip1 and the Dzip1-like protein (Dzip1L) promote primary cilium formation [21,22]. The glycogen synthase kinase 3 (GSK3) family contains two structurally similar isoforms in mammals, GSK3α and GSK3β. Beyond its function in regulating glycogen metabolism, as a multifunctional serine/threonine kinase, GSK3 also regulates the Hedgehog signaling transduction pathway [23]. A fraction of GSK3β is localized to the centrosomes, and it has increased kinase activity during the metaphase–anaphase transition [24] and is constantly active in resting cells [25]. GSK3β has also been shown to be a key component of an interlinked signaling pathway that maintains the primary cilium [26]. In this work, we investigated the mechanism of ciliogenesis during the cell cycle and found that GSK3β, Dzip1, and Rab8 co-regulate ciliogenesis by promoting ciliary membrane assembly.

## Results

### A Fraction of Dzip1 Is Localized to the Periciliary Diffusion Barrier and Concentrated at the Mother Centriole in Ciliated Cells

**Fig 1:**
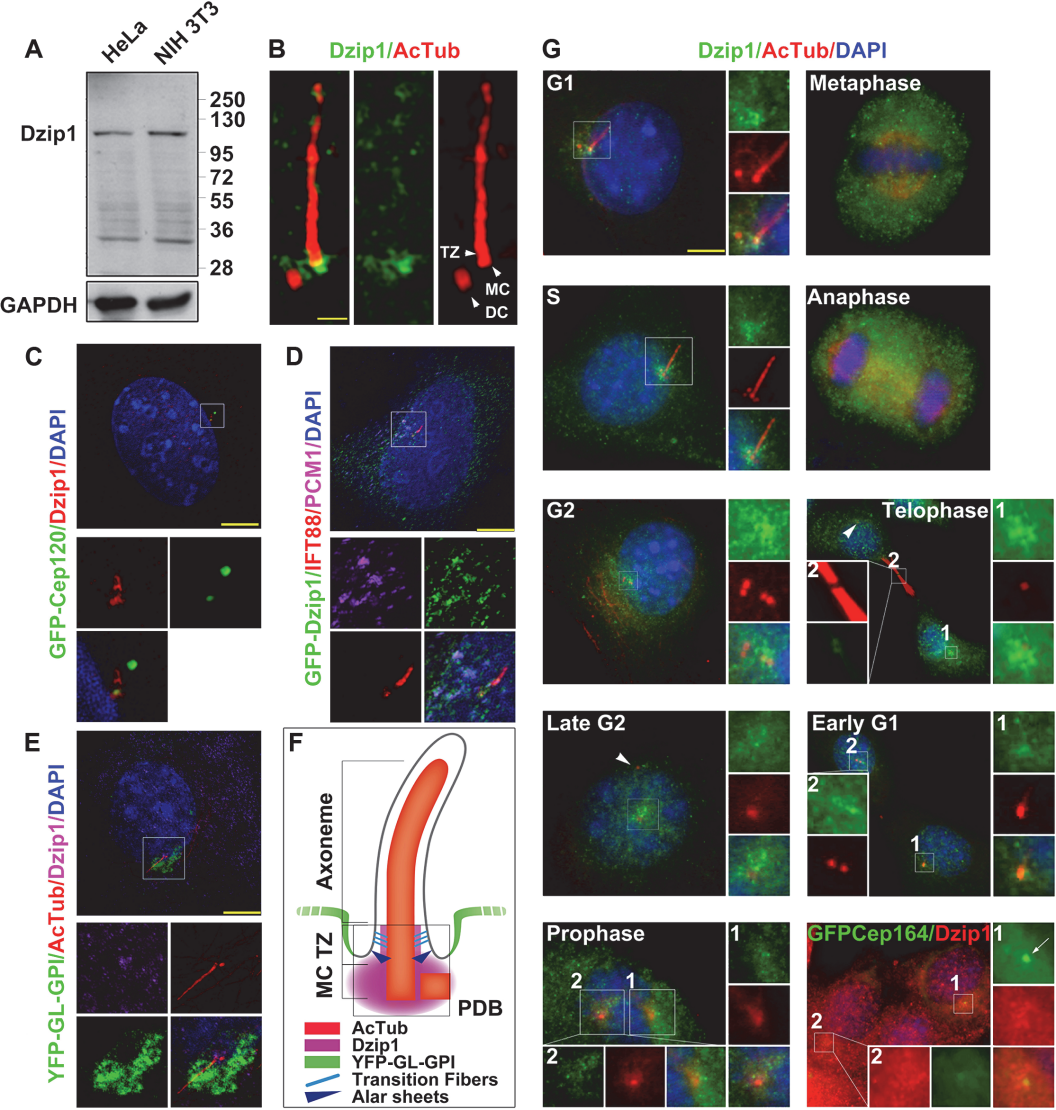
Dzip1 is localized to the periciliary diffusion barrier and concentrated at the mother centriole in ciliated cells. (A) Examination of the Dzip1 antibody. The antibody Mid2 targeting aa 594–610 of full-length human Dzip1 recognized endogenous Dzip1 at ~110 kD both in non-ciliated human (HeLa) and ciliated mouse (NIH 3T3) cells. GAPDH was set as a loading control. (B) Super-resolution microscopy showing that a fraction of Dzip1 is concentrated at the mother centriole and the PCM. G0-phase NIH 3T3 cells were immunostained with Dzip1 and acetylated α-tubulin (AcTub). DC, daughter centriole; MC, mother centriole; TZ, transition zone. Scale bar: 1 μm. (C) Super-resolution microscopy showing that Dzip1 is asymmetrically concentrated at the mother centriole. NIH 3T3 cells transfected with GFP-Cep120 were immunostained for Dzip1. Note that the brighter immunofluorescence signal of Dzip1 is partially merged with the darker signal of GFP-Cep120, and vice versa. The boxed area in the main image is magnified below it. Scale bar: 5 μm. (D) Super-resolution microscopy showing that GFP-Dzip1 is localized to the cilium base and the PCM. NIH 3T3 cells transfected with GFP-Dzip1 were immunostained for PCM1 and IFT88. Note that in addition to the co-localization of GFP-Dzip1 with PCM1 at the PCM, a portion of Dzip1 was also localized to the cilium base, labeled by IFT88. The boxed area in the main image is magnified below it. Scale bar: 5 μm. (E and F) Super-resolution microscopy showing that pericentriolar Dzip1 resides in the PDB. NIH 3T3 cells expressing the PDB marker YFP-GL-GPI were immunostained for AcTub and Dzip1. A model of localization of the indicated proteins is shown in (F). MC, mother centriole; PDB, periciliary diffusion barrier; TZ, transition zone. Scale bar: 5 μm. (G) The PCM localization of Dzip1 is dynamic in the cell cycle. NIH 3T3 cells were immunostained for Dzip1 and AcTub in the cell cycle. Note that Dzip1 was fuzzy at the centrosome in mitosis, but became evident and partially co-localized with the grandmother centriole marker GFP-Cep164 (arrow) in daughter cells in early G1 phase, during which Dzip1 also showed midbody-localization. Also note that Dzip1 was absent from one of the two centrosomes in late G2 phase and in telophase (arrowhead). Boxes labeled “1” are magnified on the right, showing the centrosomal localization of Dzip1. Scale bar: 5 μm.

To investigate the function of Dzip1, we first analyzed the protein level and the subcellular localization of endogenous Dzip1. Western blot analysis with a commercial rabbit antibody against a peptide equivalent to amino acids (aa) 594–610 of human Dzip1 (Mid2) revealed Dzip1 at ~110 kD both in non-ciliated HeLa and ciliated NIH 3T3 cells (Fig 1A). Based on immunofluorescence with this antibody, Dzip1 was found mainly in the cytoplasm, with a small amount in the nucleus. By super-resolution microscopy, we observed that the pericentriolar matrix (PCM) and the mother centriole that acts as the basal body to assemble the primary cilium were strongly stained by this antibody in G0-phase NIH 3T3 cells (Fig 1B). Similar results were obtained using an antibody against a peptide equivalent to aa 373–510 of mouse Dzip1 (Mid1; S1A and S1B Fig). Moreover, we established a stable GFP-Dzip1-expressing NIH 3T3 cell line (S1C Fig). With this cell line, we confirmed that GFP-Dzip1 was also localized to the basal body and the PCM, and found that GFP-Dzip1 was preferentially concentrated at one of the two centrioles (S1D Fig). By immunofluorescence staining the cells expressing GFP-Cep120—a protein that is asymmetrically localized to the daughter centriole [27]—we found that Dzip1 was enriched at the centriole that showed less staining for GFP-Cep120 (Fig 1C), further confirming that endogenous Dzip1 is asymmetrically enriched at the mother centriole.

Next, we investigated the precise localization of Dzip1 at the PCM by super-resolution microscopy. We observed that, in addition to its localization at the cilium base, Dzip1 was also partially co-localized with PCM1 (Fig 1D). By expressing the PDB marker YFP-GL-GPI (glycosylphosphatidylinositol-anchored glycan tagged with YFP) [5,28], we found that this portion of Dzip1 was localized inside the PDB marker (Fig 1E), indicating that the PCM-localized Dzip1 is localized to the PDB in ciliated cells (Fig 1F).

Furthermore, we examined the localization of Dzip1 during the cell cycle. We observed that Dzip1 was localized to the basal body/centrosome and the PCM in interphase, regardless of the presence or absence of a cilium. However, when the duplicated centrosomes were separated, Dzip1 showed a preferential localization on one of the two daughter centrosomes (Fig 1G), and a trace amount of it was partially co-localized with PCM1 at one of the two spindle poles (S1E Fig). This asymmetry was confirmed by GFP-Dzip1 localization in living cells (S1F Fig). In telophase/early G1 phase, Dzip1 was localized to the midbody and re-accumulated at the centrosome with priority in one of the two daughter cells (Fig 1G). By co-staining Dzip1 with the mature/mother centriole marker protein GFP-Cep164 [29,30], we confirmed that the daughter cell that inherited the grandmother centriole recruited Dzip1 earlier than the other daughter cell (Fig 1G), an outcome that is correlated with the asymmetrical ciliogenesis of the two daughter cells [31] and which suggests that Dzip1 contributes to ciliogenesis.

### Dzip1 Preferentially Binds to and Releases Rab8^GDP^ from Inhibition by GDP-Dissociating Inhibitor 2 at the Cilium Base

**Fig 2:**
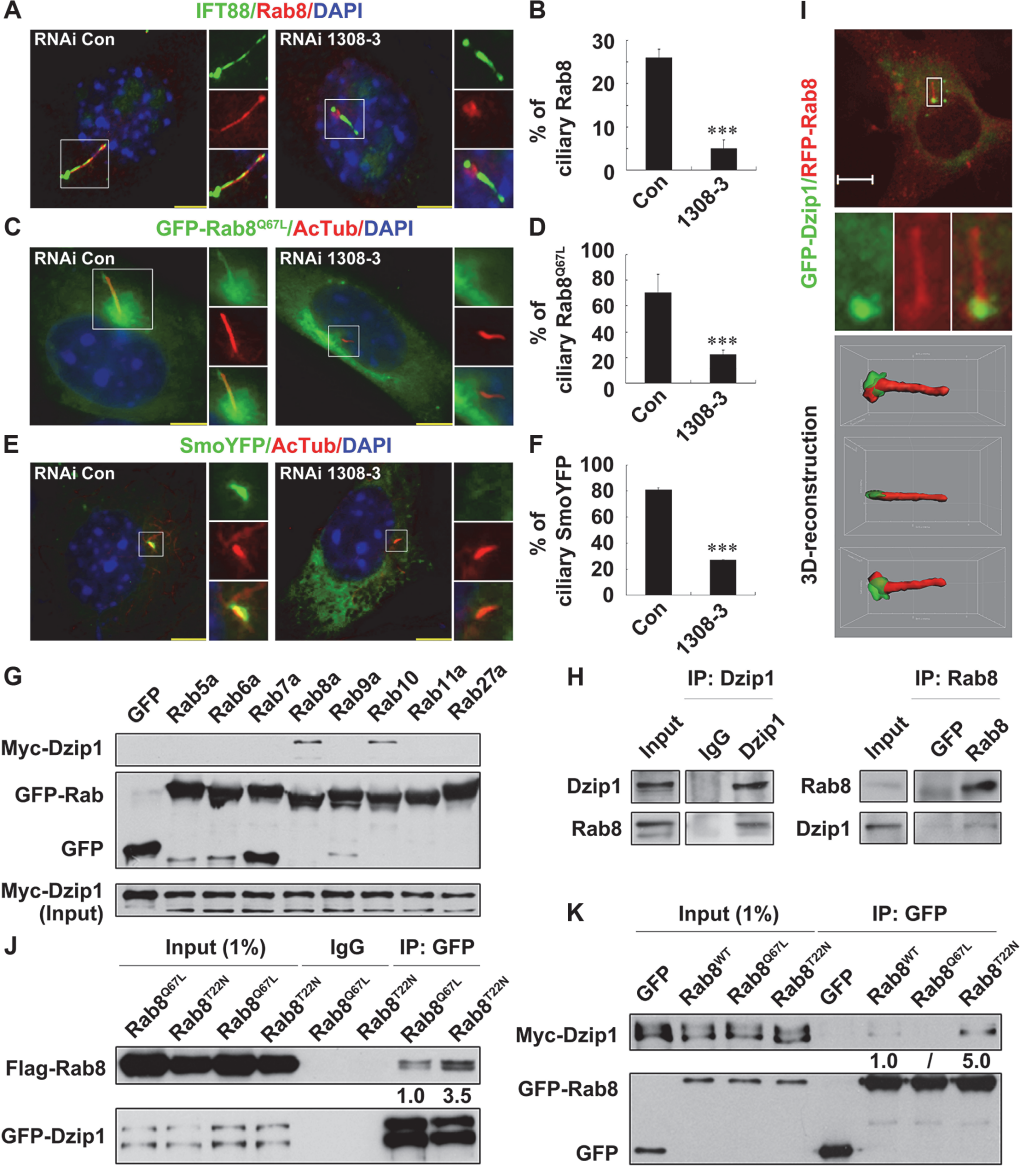
Dzip1 promotes ciliary entry of Rab8 and preferentially binds to Rab8^GDP^. (A and B) Knockdown of Dzip1 impairs ciliary localization of Rab8. RNAi control (Con) and Dzip1 stable-knockdown 1308–3 cells were arrested at G0 phase and immunostained for IFT88 and Rab8. Note that the basal body localization of Rab8 was unaffected. Scale bars: 5 μm. The values in (B) are mean ± standard deviation (SD); 100 cells were counted in each of three independent experiments. ***p < 0.001. (C and D) Rab8^GTP^ is not localized to the cilium in Dzip1-knockdown cells. The active-mimicking mutant GFP-Rab8^Q67L^ was introduced into RNAi control and RNAi 1308–3 cells, which were then immunostained for AcTub. Scale bars: 5 μm. The values in (D) are mean ± SD; 100 cells were counted in each of three independent experiments. ***p < 0.001. (E and F) Smo-YFP is not transported to the cilium in Dzip1-knockdown cells. The cells transfected with Smo-YFP were immunostained for AcTub, and the DNA was stained with DAPI. Note that, although the ciliary lengths are similar in the representative cells, ciliary Smo-YFP was much less abundant in Dzip1-knockdown cells than in control cells. Scale bars: 5 μm. The values in (F) are mean ± SD; 100 cells were counted in each of three independent experiments. ***p < 0.001. (G) Dzip1 interacts with Rab8a and Rab10. Myc-Dzip1 was co-expressed with GFP-Rab proteins or GFP alone in G0-phase cells. Immunoprecipitation (IP) was carried out using an antibody against GFP, and the immunoprecipitates were probed for the Myc tag. (H) Dzip1 interacts with Rab8. Rab8 was immunoprecipitated by Dzip1 but not by IgG in G0-phase NIH 3T3 cells (left). Dzip1 was immunoprecipitated by monoclonal antibody against Rab8 but not by GFP (right). (I) Dzip1 and Rab8 are co-localized at the cilium base. The localization of GFP-Dzip1 and RFP-Rab8 in living NIH 3T3 cells arrested in G0 phase was modeled by 3-D reconstruction (bottom). Scale bar: 5 μm. (J and K) Dzip1 preferentially binds Rab8^GDP^. The Flag-Rab8 variants were each co-expressed with GFP-Dzip1 in HEK 293T cells. Note that ~3.5-fold more Rab8^T22N^ than Rab8^Q67L^ was co-immunoprecipitated with Dzip1 (J), and ~5.0-fold more Dzip1 was co-immunoprecipitated with GFP-Rab8^T22N^ than with wild-type (WT) Rab8 (K).

To investigate the role of Dzip1 in ciliogenesis, we established two stable Dzip1-knockdown NIH 3T3 cell lines (1308–3 and 2172–1) by RNA interference (RNAi) (S2A Fig). We found that the cilium length was significantly shortened from 6.5 ± 3.2 μm in the control to 2.1 ± 0.6 and 2.6 ± 0.5 μm in the two Dzip1-knockdown cell lines, and that the percentage ciliation ratios were also decreased (S2B—S2D Fig), consistent with the concept that Dzip1 knockdown interferes with cilium assembly [8,20,21]. We further demonstrated that the defects of ciliogenesis caused by Dzip1 knockdown could be rescued by simultaneously expressing full-length RNAi-resistant Dzip1 (S2E and S2F Fig). We also observed that the localization of IFT88, which is necessary for cilium assembly [32,33], was not affected in the Dzip1-knockdown 1308–3 cells (Fig 2A), and that no IFT88 or γ-Tubulin were detectable in the GFP-Dzip1 immunoprecipitates (S3A Fig). Nevertheless, we found that ciliary Rab8 was significantly decreased in the Dzip1-knockdown 1308–3 cells (Fig 2A and 2B), although the basal body localization of Rab8 and Rabin8 was unaffected (Figs 2A and S3B). To confirm the role of Dzip1 in the entry of Rab8 into the cilium, we transiently expressed the active-mimicking mutant Rab8^Q67L^ tagged with GFP, and found that it was also unable to enter the cilium in the Dzip1-knockdown cells (Fig 2C and 2D). We further examined the localization of Smo, a membrane protein that is transported to the primary cilium depending on Rab8 [34]. Strikingly, we found that even in cells with comparable cilium lengths, Smo-YFP was localized to the cilium much less in 1308–3 cells than in the control cells (25% ± 1% versus 80% ± 2%; Fig 2E and 2F). Together, these results suggest that Dzip1 is required for the ciliary localization of Rab8 and for Rab8-mediated cargo transport to the ciliary membrane.

Next, we investigated whether Dzip1 interacts with Rab8. Using immunoprecipitation (IP) assays, we found that Dzip1 interacted strongly with Rab8 (Fig 2G and 2H). To assess the binding specificity of Dzip1-Rab8, we screened seven other Rab proteins that are involved in regulating various aspects of intracellular membrane trafficking, especially ciliogenesis. We found that Myc-Dzip1 also bound to GFP-tagged Rab10 but not Rab5a, 6a, 7a, 9a, 11a, or 27a (Fig 2G). In addition, we found that GFP-Dzip1 and RFP-Rab8 were co-localized at the cilium base in living NIH 3T3 cells (Fig 2I), and that the interaction of Dzip1 with Rab8 mainly took place at the PCM (S4A Fig). To determine whether Dzip1 discriminates between Rab8^GTP^ and Rab8^GDP^, we co-expressed Dzip1 with Rab8^Q67L^ or Rab8^T22N^ in HEK 293T cells, and found that Dzip1 bound with much more Rab8^T22N^ than Rab8^Q67L^ (Fig 2J and 2K). We also found that Rabin8, the Rab8 GEF, did not immunoprecipitate with Dzip1 (S3C Fig), suggesting that the Rab8^GDP^ bound by Dzip1 could not be converted to active Rab8^GTP^ by Rabin8. To map the region of Dzip1 that bound with Rab8, we expressed truncates of Dzip1 and analyzed their interactions with Rab8. The results showed that the C-terminus (from aa 430 to the end) of Dzip1 interacted with Rab8, of which aa 430–600 were crucial (S4B and S4C Fig). Taken together, these data demonstrate that Dzip1 preferentially binds Rab8^GDP^ via aa 430–600 at the cilium base.

**Fig 3:**
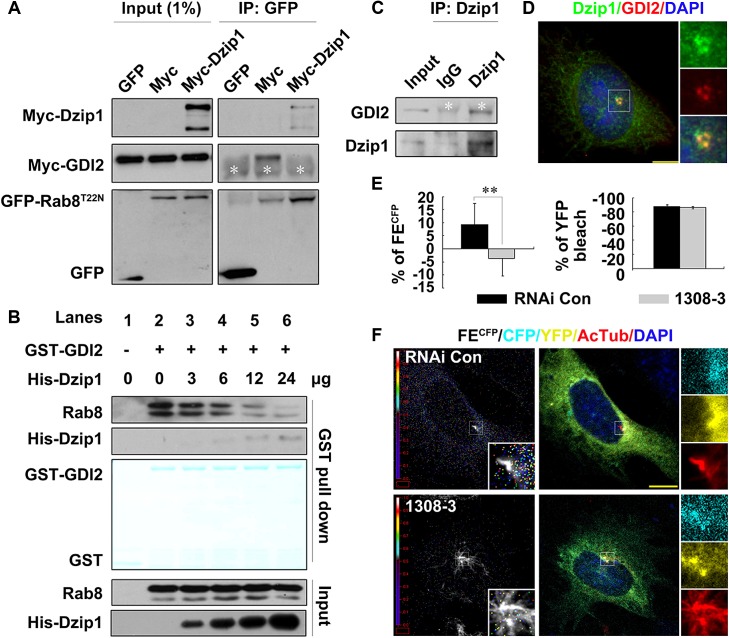
Dzip1 promotes the release of Rab8^GDP^ from GDI2 at the cilium base. (A) The binding of Myc-GDI2 to GFP-Rab8^T22N^ is abolished by expression of Myc-Dzip1. GFP-Rab8^T22N^ and Myc-GDI2 were co-expressed in HEK 293T cells with or without Myc-Dzip1 expression. White asterisks indicate the heavy chain of IgG. (B) His-Dzip1 aa 430–600 promotes the dissociation of endogenous Rab8 and GST-GDI2 in vitro. Addition of the His-Dzip1 truncate released Rab8 from GDI2 in a dose-dependent manner. The pulled-down proteins were stained with Fast Green. (C and D) Endogenous Dzip1 and GDI2 form a complex, and are co-localized at the centrosome and the PCM. Scale bar: 5 μm. (E and F) The amount of free Rab8^GDP^ at the cilium base is decreased in Dzip1-knockdown cells. CFP-RBD-YFP was introduced into G0-phase RNAi control (Con) and Dzip1-knockdown cells. The rainbow indicator shows the fluorescence emission intensity of CFP (FE^CFP^), and each colored dot in the image represents the location of FE^CFP^. The red boxes are regions selected for calibrating the background. For values in (E), 20 cells were examined. **p < 0.01. Note that the representative images show comparable CFP-RBD-YFP fluorescence intensity and YFP bleaching efficiency in both cell lines, but quantitative analysis of the acceptor-bleaching fluorescence resonance energy transfer (AB-FRET) efficiency showed a significant difference. The FE^CFP^ occurred intensively around, but not at, the basal body (labeled by AcTub) in control but not Dzip1-knockdown cells. Scale bar: 5 μm.

The enzyme cycle of Rab GTPases and their functions are regulated by not only the binding of the GTP molecule but also by GDIs, which stabilize Rab proteins in an inactive state by preventing the release of GDP from Rab proteins [15]. To understand why Dzip1 prefers to bind with Rab8^GDP^, we investigated whether the association of Rab8 with GDI2, which promotes the membrane dissociation of Rab8 [35], is regulated by Dzip1. We found that Myc-GDI2 was co-immunoprecipitated with GFP-Rab8^T22N^ from cells and that the Rab8-GDI2 interaction was abolished by overexpressing Myc-Dzip1 (Fig 3A). We also confirmed in a cell-free system that GST-GDI2, but not GST, pulled down endogenous Rab8, and when increasing amounts of His-Dzip1 aa 373–600 were added, the amount of Rab8 bound by GDI2 progressively decreased (Fig 3B). As a control, adding Myosin Va (aa 1320–1346), which interacts with Rab8 [36], had no effect on the Rab8-GDI2 dissociation (S4D Fig). Since proteins with GDF activity are able to displace GDI [16], we asked whether Dzip1 interacts with GDI2 to free Rab8^GDP^. We found that endogenous Dzip1 and GDI2 were co-localized and interacted at the basal body and the PCM (Fig 3C and 3D), and that Dzip1 also interacted with GDI2 via aa 430–600 (S4E Fig). Therefore, we concluded that Dzip1 binds with the Rab8^GDP^-GDI2 complex via its middle region and promotes the dissociation of Rab8^GDP^ from GDI2.

To directly visualize the released free Rab8^GDP^, we designed an AB-FRET assay by generating a reporter through fusing CFP and YFP tags, respectively, to the N- and C-termini of the RBD (Rab8-binding domain) of Rabin8 [14] (CFP-RBD-YFP; S5A Fig). This reporter was co-expressed with Flag-tagged Rab8^T22N^ or Rab8^Q67L^ in cells, and showed strong binding with Flag-Rab8^T22N^ but not Flag-Rab8^Q67L^ (S5B Fig), indicating that CFP-RBD-YFP mimics Rabin8 binding with Rab8^GDP^. Moreover, like endogenous Rabin8, CFP-RBD-YFP was localized to the basal body and the PCM (S5C Fig). Presumably, CFP-RBD-YFP alone may fold automatically to produce fluorescence resonance energy transfer (FRET), and the FE^CFP^ will increase after photo-bleaching of YFP (S5A Fig, panels a and b). Once bound by Rab8^GDP^, CFP and YFP will either come closer or separate from each other (S5A Fig, panels c and d), resulting in a change of FE^CFP^ after YFP photo-bleaching. Indeed, we found that a fraction of CFP-RBD-YFP was co-localized with endogenous Rab8 or Flag-Rab8^T22N^ but not with Flag-Rab8^Q67L^ in the cytoplasmic aggregates (S5D Fig), and that the FE^CFP^ in the aggregates was significantly increased after YFP photo-bleaching (S5E and S5F Fig). We also found that the more free Rab8^GDP^ was available to combine with CFP-RBD-YFP, the stronger the FE^CFP^ produced (S5E and S5F Fig). These results demonstrate that the binding of free Rab8^GDP^ with CFP-RBD-YFP separates CFP from YFP, leading to a drop in FRET from CFP to YFP and an enhancement of FE^CFP^ (S5A Fig, panel d). With this reporter, CFP-RBD-YFP, we examined control and Dzip1-knockdown cells with comparable CFP-RBD-YFP fluorescence intensity. We found that after YFP photo-bleaching, a high FE^CFP^ signal occurred around the cilium base (FE^CFP^ = 9.3% ± 8.2%) and scattered from the cilium base to the cytoplasm in control cells, but the FE^CFP^ signal at the cilium base was markedly decreased in 1308–3 cells (FE^CFP^ = −3.6% ± 6.9%; Fig 3E and 3F). The heat map showed that the FE^CFP^ signal mainly occurred at the PCM but not the centrosome (Fig 3F), indicating that Rab8^GDP^ accumulated at the PCM but was excluded from the centrosome in normal cells (Figs 3F, S4A and S4F). Taken together, these results demonstrate that Dzip1 promotes the release of Rab8^GDP^ from GDI2 at the PCM.

### Dzip1 Is Phosphorylated by GSK3β after Mitosis

**Fig 4:**
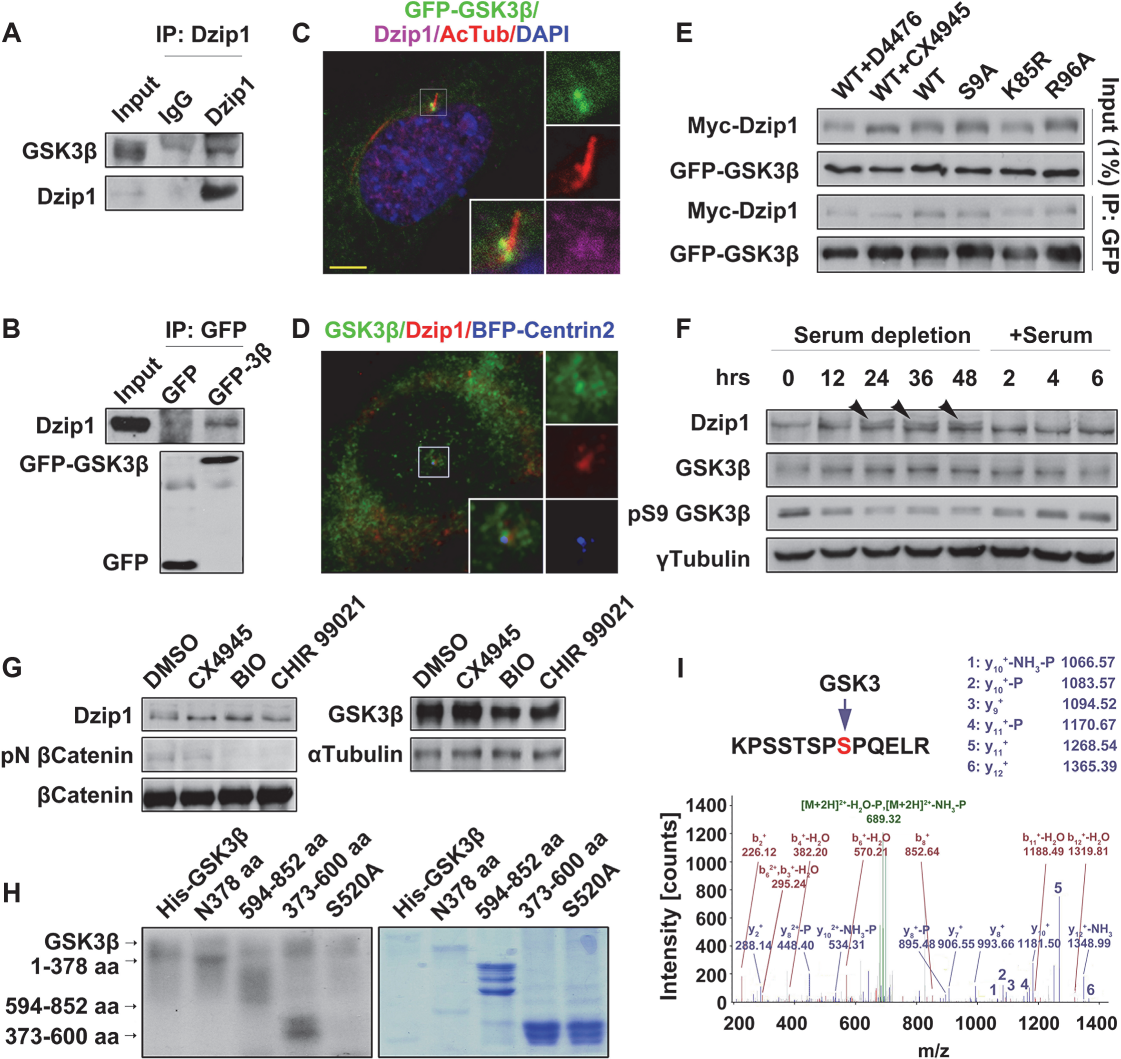
GSK3β phosphorylates Dzip1. (A and B) Dzip1 interacts with GSK3β. Endogenous GSK3β was immunoprecipitated by Dzip1 but not IgG (A), and endogenous Dzip1 was immunoprecipitated with GFP-GSK3β in HEK 293T cells (B). (C and D) Dzip1 is co-localized with GSK3β at the basal body. G0-phase NIH 3T3 cells expressing GFP-GSK3β were immunostained for Dzip1 and AcTub (C), or cells expressing BFP-Centrin2 were immunostained with GSK3β and Dzip1 (D). Scale bar: 5 μm. (E) GSK3β binds Dzip1 in a kinase-substrate interaction manner. Wild-type (WT) GFP-GSK3β and the mutants S9A, K85R, and R96A were each co-expressed with Myc-Dzip1 in G0-phase HEK 293T cells, and treated with the CK1 inhibitor D4476 or the CK2 inhibitor CX4945. Note that treatment with CX4945 but not D4476 led to a significant decrease in the extent of the up-shifted Dzip1 bands, although the binding of Dzip1 to the GFP-GSK3β variants showed no difference. The extent of the up-shifting of the Dzip1 bands was decreased in K85R-expressing cells. (F) Phosphorylation of Dzip1 is coordinated with GSK3β activation. The kinase activity of GSK3β was negatively correlated with serum stimulation in NIH 3T3 cells. Note that the up-shifted bands (arrowheads) of Dzip1 became evident after serum depletion for 24–48 h, and disappeared after serum restimulation. γ-Tubulin was set as a loading control. (G) GSK3β phosphorylates Dzip1 in vivo. In resting mouse embryo fibroblast (MEFs) treated versus not treated with GSK3 and CK2 inhibitors, the Dzip1 bands were up-shifted less in GSK3- and CK2-inhibited cells. The protein levels of total β-Catenin and GSK3β were steady, but the phosphorylated (S33/37/T41) β-Catenin specifically disappeared from GSK3-inhibited cells. α-Tubulin was set as a loading control. (H) GSK3β phosphorylates Dzip1 in vitro. Auto-phosphorylation of GSK3β (55 kD), and the phosphorylated bands of the middle (28 kD), C-terminus (36 kD), and N- terminus (50 kD) of Dzip1 are shown (left panel). Coomassie blue staining of the gel shows the loaded amounts of Dzip1 fragments (right panel). Note that the S520A mutation resulted in decreased phosphorylation of Dzip1 by GSK3β. (I) Inhibition of GSK3 by BIO causes loss of phospho-S520 in Dzip1.

Since the PCM localization of Dzip1 during the cell cycle is prior to ciliogenesis and Dzip1 plays a positive role in the regulation of ciliogenesis by promoting the dissociation of Rab8^GDP^-GDI2 and ciliary entry of Rab8 as shown above, we set up experiments to test how Dzip1-mediated ciliogenesis is regulated after mitosis. By comparing the expression of Dzip1 in NIH 3T3 cells arrested at G0 phase and prometaphase through Western blot analysis, we found that Dzip1 was up-shifted in G0-phase cells and that these up-shifted bands were sensitive to λ phosphatase (S6A Fig), indicating that a portion of Dzip1 is phosphorylated in resting cells. In searching for the kinase(s) responsible for the phosphorylation of Dzip1, we realized that GSK3β might be the best candidate because of its centrosomal localization, its increasing activity from metaphase to anaphase, and its high activity in the absence of growth factors [37–39] (S6B Fig). Indeed, we found that GSK3β was immunoprecipitated by Dzip1, and vice versa (Fig 4A and 4B), and that the two proteins were co-localized at the basal body (Fig 4C and 4D).

To determine whether GSK3β interacts with Dzip1 in a kinase-substrate-binding manner, we assessed the binding intensity of wild-type and mutant GFP-GSK3β with Myc-Dzip1. We found that the binding of Myc-Dzip1 with the mutant GSK3β^R96A^—which cannot phosphorylate primed substrates but retains intact kinase activity for the unprimed substrates [37,40]—showed no decrease in comparison with wild type, the constantly active mutant GSK3β^S9A^, or the kinase-dead mutant GSK3β^K85R^ [37]. Furthermore, inhibition of CK2 by CX4945 caused a remarkable reduction in the extent of Dzip1 band up-shifting, whereas inhibition of CK1 by D4476 did not change the up-shift of Dzip1 compared with untreated cells (Fig 4E), indicating that Dzip1 is phosphorylated by CK2 as previously reported [41]. Interestingly, both CK2-phosphorylated and-nonphosphorylated Myc-Dzip1 equally bound to GFP-GSK3β (Fig 4E), suggesting that the phosphorylation of Dzip1 by CK2 does not change the Dzip1-GSK3β interaction. Notably, expression of GSK3β^K85R^ yielded a weaker up-shift of Dzip1 than expression of wild type or other mutants (Fig 4E). Together, these results indicate that Dzip1 is a substrate of GSK3β independent of priming phosphorylation by CK1 and CK2.

Next, we investigated Dzip1 phosphorylation during cell cycle exit and re-entry. We found that depletion of serum over a time period of 48 h caused a mild increase in total GSK3β at the protein level and enhancement of its kinase activity, as indicated by the phosphorylation decrease of GSK3β at S9 [37] (Fig 4F). In contrast, when serum was reintroduced, GSK3β kinase activity decreased (Fig 4F). We also found that the up-shifted bands of Dzip1 became evident after 12 h of serum depletion and more pronounced in the following 36 h. However, the up-shifted bands rapidly disappeared during the G0 phase–G1 phase transition (Fig 4F). Moreover, we assessed the phosphorylation status of Dzip1 in the presence of the GSK3 inhibitors BIO and CHIR99021 or the CK2 inhibitor CX4945 in resting immortalized MEFs. The efficiency of GSK3 inhibition was monitored by de-phosphorylation of its canonical substrate β-Catenin. The up-shifted bands of Dzip1 disappeared in cells treated with the CK2 inhibitor but not in control cells, as previously reported [41], and weakened under treatment with the GSK3 inhibitors, further supporting that GSK3β phosphorylates Dzip1 (Fig 4G).

To demonstrate Dzip1 phosphorylation by GSK3β, an in vitro kinase assay was performed using the kinase His-GSK3β with the wild-type Dzip1 truncates aa 1–378, aa 373–600, and aa 594–852. The results showed that His-GSK3β phosphorylated all three wild-type truncates (Fig 4H). The specificity of the in vitro phosphorylation assay was confirmed by the findings that the truncate aa 373–600 was neither phosphorylated by His-GSK3β^K85R^ nor under the inhibition of GSK3 (S6C Fig), and that neither His-Rab8 nor GST-GDI2 was phosphorylated by GSK3β in a parallel experiment (S6D Fig). To characterize the phosphorylation site(s) on Dzip1 by GSK3β, GFP-Dzip1 was immunoprecipitated and subjected to identification of phospho-peptide by mass spectrometry analysis. The results showed that S520 on Dzip1 was phosphorylated (Fig 4I) and that this phosphorylation disappeared in the cells treated with the GSK3 inhibitor BIO. This site was also confirmed by the in vitro kinase assay, which showed that the S520A mutant of the truncate aa 373–600 was unable to be phosphorylated by the kinase (Fig 4H). Taken together, these data demonstrate that Dzip1 is phosphorylated at S520 by GSK3β in G0-phase cells.

### Phosphorylation of Dzip1 by GSK3β Promotes the Release of Rab8^GDP^ from GDI2

**Fig 5:**
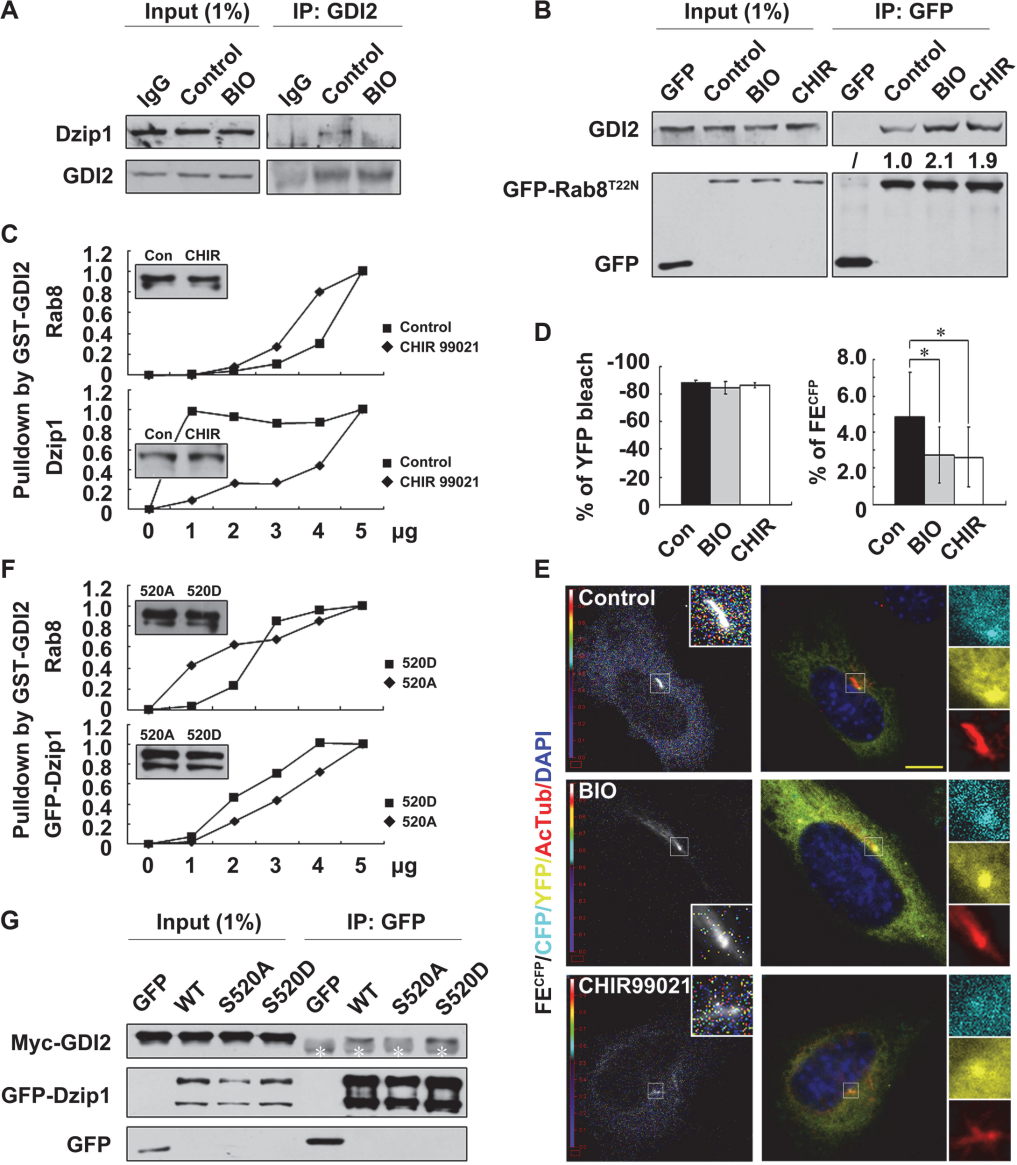
Phosphorylation of Dzip1 by GSK3β promotes dissociation of Rab8^GDP^ from GDI2. (A) The binding of Dzip1 with GDI2 is prevented by inhibition of GSK3. G0-phase NIH 3T3 cells were treated or not treated with the GSK3 inhibitor BIO, and endogenous Dzip1 and GDI2 were analyzed. (B) The binding of Rab8^GDP^ with GDI2 is increased by inhibition of GSK3. G0-phase HEK 293T cells expressing GFP-Rab8^T22N^ were treated or not treated with the GSK3 inhibitors BIO or CHIR99021 (CHIR), and endogenous GDI2 was analyzed. The quantified band intensities are labeled. (C) The binding of GDI2 to Dzip1 is decreased, but to Rab8 is increased, by inhibition of GSK3. Endogenous Dzip1 and Rab8 were pulled down from G0-phase NIH 3T3 cells treated with the GSK3 inhibitor or from control (Con) cells by increasing amounts of GST-GDI2. The maximum amounts of the pulled-down proteins were defined as having a binding affinity of 1.0, and the amounts of the indicated proteins at each point were normalized to the maximum amounts. The results represent two independent assays. (D and E) The amount of free Rab8^GDP^ at the basal body is decreased by inhibition of GSK3. G0-phase NIH 3T3 cells expressing the fusion protein CFP-RBD-YFP were treated with the GSK3 inhibitors BIO or CHIR99021 or not treated (control). The values shown are mean ± SD, from 20 cells for each group (D). The calculated region was manually selected at the cilium base based on strong accumulation of the CFP-RBD-YFP signal. *p < 0.05. Scale bar: 5 μm. Note that the images from all treatment situations showed comparable CFP-RBD-YFP fluorescence intensity but different FE^CFP^ distributions in quantitative analysis. The FE^CFP^ was highest around the basal body (labeled by AcTub staining) in the control cell, but was largely eliminated in GSK3-inhibited cells. The rainbow indicator in the heat map shows the FE^CFP^, and each colored dot in the image represents the appearance of FE^CFP^. The red boxes indicate the areas selected for calibrating background (E). (F) Expression of Dzip1^S520A^ leads to increased binding of Rab8 to GDI2. The GFP-Dzip1 mutants and endogenous Rab8 were pulled down from G0-phase HEK 293T cells by increasing amounts of GST-GDI2. The maximum amounts of the pulled-down proteins were defined as having a binding affinity of 1.0, and the amounts of the indicated proteins at each point were normalized to the maximum amounts. The results represent two independent assays. (G) Phosphorylation of Dzip1^S520^ increases its binding to GDI2. GFP alone or wild-type (WT) GFP-Dzip1 or S520 mutants were co-expressed with Myc-GDI2 in G0-phase HEK 293T cells, followed by IP assay. White asterisks indicate the heavy chain of IgG.

Due to the importance of aa 430–600 of Dzip1 in its binding with Rab8-GDI2, we tested whether phosphorylation at S520 within this region influences this interaction. We found that inhibition of GSK3 markedly decreased the binding affinity of Dzip1 for GDI2 (Figs 5A and S6E) and significantly increased the binding affinity of Rab8^GDP^ for GDI2 (Fig 5B), suggesting that GSK3 is involved in the regulation of Rab8^GDP^ dissociation from GDI2 via regulating the binding of Dzip1 with GDI2. To further confirm this notion, we performed a semi-quantitative GST-GDI2 pulldown assay. We found that as increasing amounts of GST-GDI2 were added, the pulled-down amounts of endogenous Dzip1 and Rab8 showed different trends: the rate of Dzip1-GDI2 binding was much higher in control cells than in the cells treated with CHIR99021 (i.e., GDI2 was much more quickly saturated by Dzip1 in control cells than in CHIR99021-treated cells), whereas the rate of Rab8-GDI2 binding was lower in control cells than in CHIR99021-treated cells (Fig 5C). To directly compare the amounts of Rab8^GDP^ released from GDI2 with and without GSK3 regulation, we measured FE^CFP^ in cells expressing comparable amounts of CFP-RBD-YFP. We found that the efficiencies of YFP photo-bleaching at the basal body were steadily decreased by ~85% both in control cells and the cells treated with the GSK3 inhibitor. However, the FE^CFP^ signal after YFP photo-bleaching was lower in cells treated with BIO (3.3% ± 2.1%) and CHIR99021 (2.6% ± 1.7%) than in control cells (4.8% ± 2.8%; Fig 5D and 5E). Consistently, the binding of Flag-Rab8^T22N^ to the FE^CFP^ reporter CFP-RBD-YFP was decreased under GSK3 inhibition (S6G Fig).

To understand whether the decreased Dzip1-GDI2 and increased Rab8-GDI2 binding with GSK3 inhibition was due to the phosphorylation of Dzip1 at S520, we compared the amounts of Dzip1 mutants and endogenous Rab8 pulled down by GDI2. We found that the rate of Rab8-GDI2 binding was much lower in cells expressing Dzip1^S520D^ than in those expressing Dzip1^S520A^, and the rate of Dzip1^S520D^-GDI2 binding was higher than that of Dzip1^S520A^–GDI2 (Fig 5F). Coinciding with this, when wild-type or mutant GFP-Dzip1 was co-expressed with Myc-GDI2 in G0-phase HEK 293T cells, we found that the mutant Dzip1^S520A^ showed much less binding affinity for GDI2 than wild-type Dzip1 and the mutant Dzip1^S520D^ (Fig 5G). Taken together, these results demonstrate that the phosphorylation of Dzip1 at S520 by GSK3β promotes the dissociation of Rab8^GDP^-GDI2.

### GSK3β Regulates Ciliogenesis by Phosphorylating Dzip1 after Mitosis

**Fig 6:**
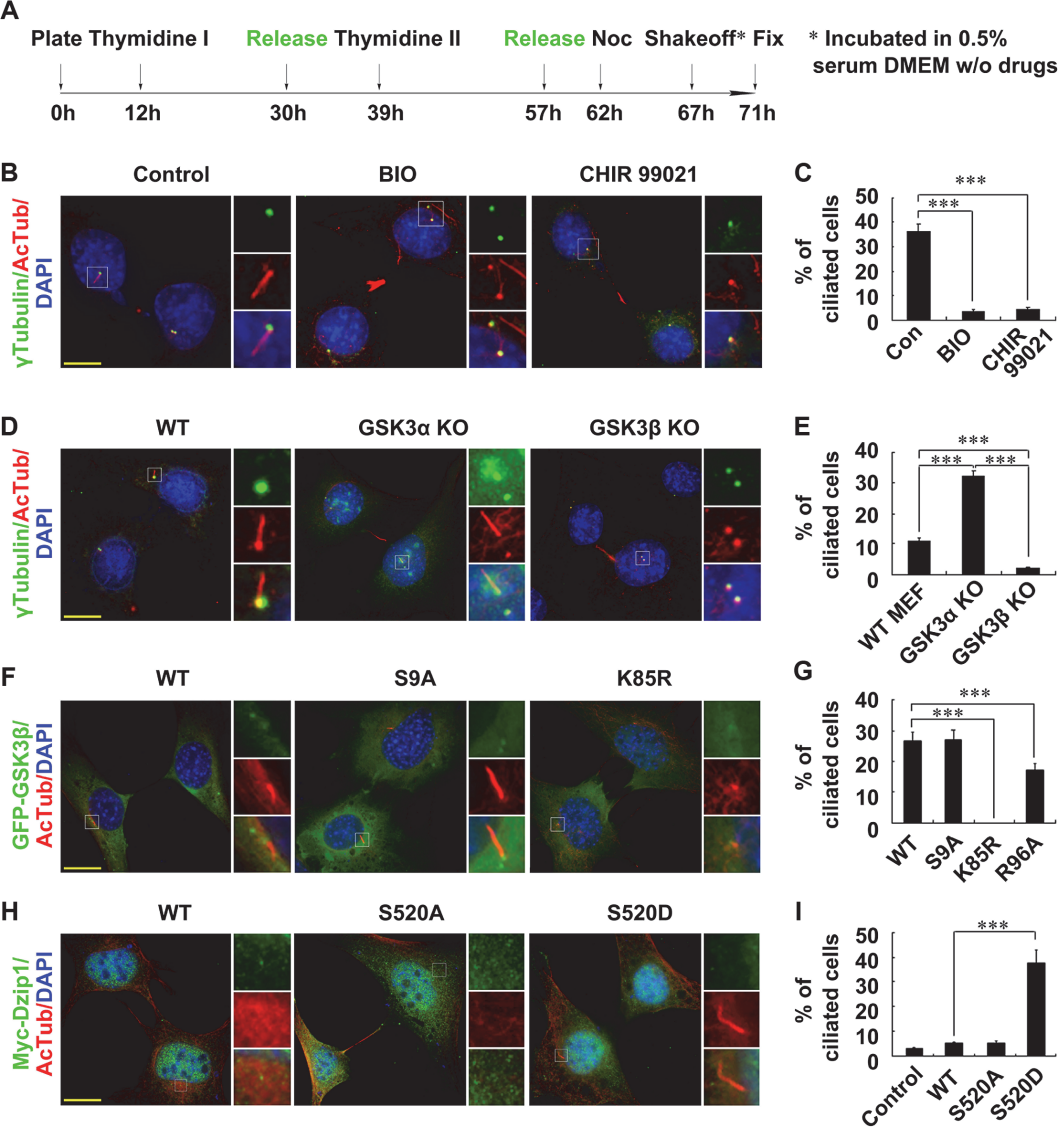
Dysfunction of GSK3β disrupts ciliogenesis. (A) Schematic model of the acquisition of synchronized mitosis–G0 phase NIH 3T3 cells, treated with double thymidine block, nocodazole (Noc), and the indicated GSK3 inhibitors. (B and C) Inhibition of GSK3 impairs ciliogenesis. Representative images of ciliated cells during the mitosis–G0 phase transition without (Con) or with BIO or CHIR99021 treatment. Cells were immunostained for γ-Tubulin and AcTub. The DNA was stained with DAPI. Ciliation ratios for (B) are shown in (C). (D and E) Knockout (KO) of GSK3β, but not GSK3α, abolishes ciliogenesis. Wild-type (WT), GSK3α^−/−^, and GSK3β^−/−^ MEFs during the mitosis–G0 phase transition were immunostained with γ-Tubulin and AcTub. The DNA was stained with DAPI. Ciliation percentage ratios for (D) are shown in (E). (F and G) The kinase activity of GSK3β is required for ciliogenesis. GSK3β^−/−^ MEFs were transfected with wild-type GFP-GSK3β or mutant GFP-GSK3β S9A, K85R, or R96A and immunostained with AcTub. The DNA was stained with DAPI. Representative images are shown in (F), and the percentage ciliation ratios are shown in (G). Note that the wild-type and S9A fully, and R96A partially, rescued the ciliogenesis defect, whereas K85R did not. (H and I) A phosphorylation-mimicking mutant of Dzip1 rescues the ciliogenesis defect in GSK3β^−/−^ cells. GSK3β^−/−^ MEFs were transfected with wild-type Myc-Dzip1, mutant S520A, or S520D, and immunostained with AcTub. The DNA was stained with DAPI. Representative images are shown in (H), and the percentage ciliation ratios are shown in (I). The values in (C), (E), (G), and (I) are mean ± SD; 50 pairs of daughter cells were counted in each of three independent experiments. ***p < 0.001. Scale bars in (B), (D), (F), and (H): 5 μm.

To understand the function of Dzip1 phosphorylation by GSK3β, we then investigated the effect of GSK3 inhibition on post-mitotic NIH 3T3 cells, and found that the percentage ciliation ratios of the post-mitosis daughter cells were dramatically decreased to 3.5% ± 1.0% with BIO treatment and to 4.5% ± 0.8% with CHIR99021 treatment, compared with 36% ± 3.1% in control cells at the 71-h time point after cell synchronization (i.e., after serum starvation for 4 h) (Fig 6A–6C). The reduced percentage ciliation ratio with CHIR99021 treatment was not due to cytokinesis delay or failure, since less than 20% of the cells serum-starved for 24 h were ciliated with this treatment, whereas ~70% of the untreated serum-starved control cells were ciliated at the same time point (S7A and S7B Fig). Moreover, the lengths of the assembled cilia in BIO- and CHIR99021-treated cells were markedly shorter (2.2 ± 1.0 μm and 1.8 ± 1.2 μm, respectively) than that in control cells (4.9 ± 1.0 μm; S7C Fig).

As the kinase domains of GSK3α and GSK3β are highly homologous [37], we wondered whether GSK3α or GSK3β or both are responsible for regulating ciliogenesis. By separately investigating ciliogenesis in immortalized MEFs from wild-type and GSK3α- and GSK3β-knockout mice [25] (S7D Fig), we found that only GSK3β^−/−^ cells exhibited ciliogenesis failure after exit from mitosis (Fig 6D and 6E). Notably, although wild-type GSK3β and its mutants S9A and K85R showed identical basal body localization, only wild type and the mutant S9A, but not K85R, efficiently rescued ciliogenesis failure in post-mitotic and in resting GSK3β^−/−^ cells (Figs 6F and 6G, S7E and S7F), demonstrating that the enzymatic activity of GSK3β is required for ciliogenesis in post-mitotic cells. We also used R96A to rescue ciliogenesis failure in GSK3β^−/−^ cells and found that it could partially rescue ciliation in post-mitotic and in resting cells (Figs 6F and S7F), suggesting that direct phosphorylation of the substrate(s) by GSK3β is involved in cilium assembly.

Finally, we set up an experiment to determine whether the phosphorylation of Dzip1 by GSK3β regulates ciliogenesis. By expressing the wild-type Dzip1, the nonphosphorylatable mutant Dzip1^S520A^ (by GSK3β), or the phosphorylation-mimicking mutant Dzip1^S520D^ in GSK3β^−/−^ cells, we found that the expression of Dzip1^S520D^ markedly elevated the ciliogenesis ratio from 3.0% ± 0.3% to 38% ± 3.2%, whereas wild-type Dzip1 and the mutant Dzip1^S520A^ had no ability to rescue ciliogenesis in GSK3β^−/−^ cells (Fig 6H and 6I). Consistently, Dzip1^S520D^, but not Dzip1^S520A^, elevated the ratio of cilium-localized Rab8 in GSK3β^−/−^ cells, and rescued the cilium defect in 1308–3 cells (S7G—S7J Fig). Taken together, these results demonstrate that Dzip1 phosphorylation by GSK3β is required for ciliogenesis after exit from mitosis.

## Discussion

Several lines of evidence suggest that the PDB plays an important role in regulating the transport of cilium proteins, but how the PDB proteins execute their functions remains largely unknown. The present work revealed that a PDB-localized protein, Dzip1, functions as a GDF that promotes Rab8^GDP^ dissociation from GDI2 at the PCM through binding with GDI2, and that this binding is positively regulated by the activation of GSK3β during ciliogenesis. The Rab proteins bound with GDP bind GDI with very strong affinity, whereas the membrane-bound GDFs promote the release of the GDP-bound Rab proteins from GDI, thereby facilitating the association of Rab proteins with relevant membranes [15]. Given that GEFs for Rab proteins cannot directly bind with Rab^GDP^-GDI complexes [16], we propose that, in the present case, the Dzip1-mediated Rab8-GDI2 dissociation is a prerequisite for Rab8 activation by its GEF Rabin8.

So far, two proteins besides Dzip1 have been identified as GDFs. One is Yip3/PRA1, a protein with GDF activity and without GEF activity, which promotes the dissociation of Rab9 from GDI and facilitates Rab9 insertion on the membrane [42,43]; the other is SidM/DrrA, which retains both GDF and GEF activities and promotes the recruitment of Rab1 to Legionella-containing vacuoles during host-cell infection [44]. Dzip1 contains a Zn-finger in its N-terminus, like another Zn-finger-containing protein, Mss4/Dss4, an evolutionarily conserved Rab chaperone factor that promotes nucleotide release from exocytic Rab GTPases at a very low rate [45–47]. However, Dzip1 shows no sequence similarity to Mss4. Dzip1 specifically bound with Rab8 and Rab10 in our assays, unlike the broad binding of Mss4 with a number of Rab members [48]. Considering that Dzip1 and Rab8 are only co-localized at the centrosome and that Rabin8 could exchange the nucleotide for Rab8 at the centrosome [14,17], we conclude that Dzip1 functions more like a GDF specific for Rab8 than like a GEF at the centrosome. Moreover, it is noteworthy that Dzip1 also interacts with Rab10, the closest Rab8 paralogue, which is associated with the primary cilium and co-localizes and interacts with members of the exocyst complex in renal epithelial cells [49]. However, whether this interaction is due to the high sequential similarity between Rab8 and Rab10 (identity 76%) or the involvement of Dzip1 in regulating Rab10 activity remains so far unclear.

Once dissociated from GDI2, Rab8^GDP^ is ready for a subsequent switch into Rab8^GTP^ under the regulation of Rabin8. In the absence of Dzip1, Rab8—as well as its active-mimicking form Rab8^Q67L^—did not enter cilium, although it was still localized to the basal body. Dzip1 interacts with Cep164 [22], and loss of Cep164 leads to impaired vesicle docking at the mother centriole and failed ciliary localization of the wild-type Rab8 but not the mutant Rab8^Q67L^ [50]; therefore, we propose that in addition to regulating Rab8 activation, Dzip1 also regulates the translocation of Rab8^GTP^ across the transition zone or PDB downstream of Cep164. As ciliogenesis occurs first in daughter cells that have inherited the grandmother centriole [31], which is persistently capped by a primary cilium-derived vesicle during mitosis [30], our finding that the grandmother-centriole-containing daughter cell recruited Dzip1 to its centrosome earlier than the other daughter cell indicates that Dzip1 plays a crucial role in regulating this asymmetrical ciliogenesis.

In the absence of growth factors, the kinase activity of GSK3β is de-repressed. Our findings suggest that once the amount of active GSK3β reaches a threshold after mitosis, GSK3β regulates the release of Rab8^GDP^ from GDI2 at the cilium base by phosphorylating Dzip1 at S520 to promote ciliogenesis. However, when cells are growing in a nutrient-rich environment, GSK3β is inactivated by its inhibitory kinases and is unable to phosphorylate Dzip1. Therefore, the lack of free Rab8^GDP^ may fail to support the generation of Rab8^GTP^ for ciliogenesis, leading to the disappearance of cilia from proliferating cells. Interestingly, in contrast to the viability of mice lacking GSK3α, knockout of GSK3β is lethal due to either hepatic apoptosis [25] or hypertrophic myopathy induced by cardiomyocyte hyperproliferation [51]. As the primary cilium is proposed as a “brake lever” of the cell cycle [52], negatively regulating cell cycle re-entry [53,54], the finding that GSK3β knockout induces cardiomyocyte hyperproliferation suggests a link between GSK3β and cilium assembly. Indeed, it has been shown that GSK3β is required for cilium maintenance via regulation of the microtubule-stabilizing protein pVHL [26,55]. Our present findings further extend the function of GSK3β in the regulation of ciliogenesis, support a positive role of GSK3β in the regulation of cilium assembly, and may have implications for understanding why rapidly-proliferating cells (such as cancer cells) often lose their cilia.

## Materials and Methods

### Antibodies and Reagents

The CK1 inhibitor D4476 (Tocris Bioscience, 2902), rabbit anti-GSK3β (Santa Cruz Biotechnology, sc-9166), β-Catenin (BD, 610154), phosphorylated GSK3β (S9, Cell Signaling Technology, #93365), and phosphorylated β-Catenin (N3, Cell Signaling Technology, #9561) were kind gifts from Dr. Wei Wu (Tsinghua University). The CK2 inhibitor CX4945 (Sequoia Research Products, SRP04555c) was kindly provided by Dr. Seong H. Kim (Korea University of Science and Technology). The GSK3 inhibitors BIO (Tocris Bioscience, 3194) and CHIR99021 (Tocris Bioscience, 4423) were from Tocris Bioscience. Rabbit anti-Dzip1 polyclonal antibody (re-named Mid2) was from Abgent (AP8926c). Mouse anti- Dzip1 and anti-GDI2 polyclonal antibodies were produced from mice immunized with aa 370–510 (named Mid1) of mouse Dzip1 and with the full-length of human GDI2, respectively. Rabbit anti-IFT88 (Proteintech, 13967-1-AP), Rabin8 (Proteintech, 12321-1-AP), PCM1 (Santa Cruz Biotechnology, sc-67204), GDI2 (Santa Cruz Biotechnology, sc-133939), histone H3 pS10 (Abcam, ab47297), mouse anti-GAPDH (Proteintech, 60004-1-lg), GFP (MBL International, M048-3), Flag (MBL International, PM020), Myc (Sigma, M4439-100UL), γ-Tubulin (Sigma, T6557), α-Tubulin (Sigma, T5168), AcTub (Sigma, T7451), and Rab8 (BD, 610844) were from the indicated companies and were used according to the standard protocols provided. All animal experiments were performed according to the approved guidelines.

### Cell Culture, Synchronization, Transfection, and Establishment of Stable Cell Lines

Cells were cultured at 37°C in Dulbecco’s modified Eagle’s medium containing 10% fetal bovine serum under standard conditions. Wild-type and GSK3α- and GSK3β-knockout MEF cells were kindly provided by Prof. Jim Woodgett (University of Toronto). To obtain mitotic NIH 3T3 cells, the cells were double-treated with thymidine to synchronize them in early S phase, and then released into medium containing nocodazole (50 nM) for 8 h. To arrest NIH 3T3 cells at the mitosis–G0 transition, the round-up cells after nocodazole treatment were shaken off and reseeded onto coverslips in fresh medium containing 0.5% serum with or without the GSK3 inhibitors BIO (2 μM) or CHIR99021 (10 μM). Transient cDNA transfections were carried out on cells using the Megatrans transfection reagent (Origene, TT200002) according to the manufacturer’s instructions. To select cell lines stably expressing GFP-Dzip1 or with Dzip1 knockdown, NIH 3T3 cells were split 24 h after vector transfection and treated with G418 or Puromycin (Sigma) for 2 wk until the clones became macroscopic. Stable clones were verified by Western blot assay.

### Molecular Cloning and RNA Interference

Human GSK3β (BC000251), GDI2 (BC005145), Rabin8 (BC059358), and mouse Dzip1 (BC098211) were each cloned from the cDNA libraries of the human B cell and mouse embryo brain (E14). Mutagenesis was conducted using standard molecular approaches. The DNA fragments for expressing the short hairpin RNAs that target the base pair positions 1308–1327 and 2172–2191 of the mouse Dzip1 gene were generated by annealing the following pairs of oligonucleotides (only sense sequences are shown): 5′-GATCCCCCTGAAAGGGACTCCTTTAATTCAAGAGATTAAAGGAGTCCCTTTCAGTTTTTA-3′ and 5′-GATCCCCCTGACAGGAACCTCCATTATTCAAGAGATAATGGAGGTTCCTGTCAGTTTTTA-3′, and the annealed oligonucleotides were then ligated into the pSuper-RetroPuro plasmid (OligoEngine). Transfection of these plasmids using Megatrans (Origene) was performed according to the manufacturer’s instructions.

### Basal Body Purification and Sucrose Gradient Ultracentrifugation

Basal body purification was carried out as previously described [56]. A 30%–70% sucrose gradient was prepared in PIPES buffer (0.1% Triton X-100, 10 mM PIPES [pH 7.2], 0.2% EtSH). The collected basal bodies were then loaded onto a gradient containing 70%, 50%, 40%, and 30% sucrose from bottom to top, and centrifuged at 120,000*g* for 1 h. Fractions (~100 μl each) were carefully collected from the top, mixed with sample buffer, and analyzed by standard Western blotting procedures.

### Immunoprecipitation and Western Blotting

For IP assays, NIH 3T3 or HEK 293T cells transfected with the indicated plasmids were lysed using IP buffer (0.1% NP-40, 50 mM HEPES [pH 7.00], 125 mM NaCl, 10% glycol, 0.5 mM PMSF) on ice for 15 min. Lysates were centrifuged at 12,000 rpm for 15 min, and the supernatants were incubated with the primary antibody-coated beads for 1.5 h at 4°C on a rotator. After six washes with IP buffer, the beads were collected and the bound proteins were analyzed by standard Western blotting [8]. The intensity of the indicated bands was quantified using ImageJ (US National Institutes of Health).

### Immunofluorescence Microscopy and Live-Cell Imaging

Immunofluorescence was performed as previously described [8]. Briefly, cells were fixed in 4% paraformaldehyde/phosphate buffer solution (PBS) followed by extraction in 0.2% Triton X-100/PBS, or cells were fixed in cold methanol. The cells were sequentially immunostained with the indicated primary and secondary antibodies. DNA was stained with DAPI, and the coverslips were mounted with Mowiol (Sigma). For live-cell imaging of cells co-transfected with GFP-Dzip1 and RFP-Rab8, the original images were collected at 2-μm thickness for five layers every 20 min. For image acquisition and processing, Zen 2009 Light Edition and Velocity (6.1.1) were respectively used for management of the confocal immunofluorescence microscope (Carl Zeiss, LSM-710NLO and DuoScan), and the UltraView Vox spinning disc confocal microscope (PerkinElmer). Super-resolution microscopy was performed with a 3-D structured illumination microscope (Nikon, N-SIM), equipped with 405-, 488-, and 594-nm lasers, electron-multiplying CCD cameras (iXon DU-897E, 512 × 512, 16 μm × 16 μm), and a CFI SR Apo TRIF 100× (1.49NA) oil objective (Nikon). The 3-D images were then reconstructed using the NIS-Elements AR software package (Nikon).

### Acceptor-Bleaching Fluorescence Resonance Energy Transfer

The N- and C-termini of the RBD (Rab8-binding domain) of Rabin8 were fused with CFP and YFP, respectively. This fusion protein was introduced into G0-phase NIH 3T3 cells. For fixed cells, only those that displayed apparent basal body localization of CFP-RBD-YFP were selected for AB-FRET assay. Regular immunofluorescence images (all channels) were taken first to show common features of the selected cell. Before acceptor (YFP) bleaching, CFP and YFP channel images were respectively captured three times to calculate the mean fluorescence intensity of *I*_CFP before_ and *I*_YFP before_. At the time point of photo-bleaching, a 514-nm laser at 100% intensity was activated 400 times to bleach the emission of YFP in the selected regions (boxed), after which the fluorescence intensity of YFP (*I*_YFP after_) steadily dropped over 80%. After photo-bleaching of YFP, at the following seven time points (with the same intervals), the fluorescence intensities of CFP and YFP were each measured to calculate the means of *I*_CFP after_ and *I*_YFP after_. The fluorescence efficiency of FE^CFP^ was calculated as *I*_FRET_ = (*I*_CFP after_ − *I*_CFP before_)/*I*_CFP before_. Heat maps were automatically generated according to the manual of image processing (Carl Zeiss, LSM-710NLO and DuoScan).

### Protein Expression, Purification, and In Vitro Kinase Assay

His-tagged Dzip1 fragments, His-GSK3β, or GST-GDI2 were expressed and purified from *Escherichia coli*. BL21 with extraction buffer (50 mM NaH_2_PO_4_, 300 mM NaCl, 10 mM imidazole [pH 8.0]) or PBS. Briefly, the bacterial pellet was ultrasonically lysed, and the supernatant was incubated with His60 Ni Superflow resin or GST resin (Clontech) at 4°C for 1 h. Nonspecific binding to the resin was eliminated by three washes with the wash buffer (50 mM NaH_2_PO_4_, 300 mM NaCl, 20 mM imidazole [pH 8.0]) or PBS, and the indicated proteins were eluted with elution buffer (50 mM NaH_2_PO_4_, 300 mM NaCl, 250 mM imidazole [pH 8.0], for His-tagged proteins; 10 mM reduced glutathione in 50 mM Tris-HCl [pH 8.0], for GST-tagged proteins) and substituted by PBS buffer. For purification of the aa 1–378 fragment of Dzip1 from inclusion bodies, 8 M urea denaturation and graded re-naturation with a dialysis bag (8 M–4 M–3 M–2 M–1 M in PBS with 3 mM reduced glutathione, 0.3 mM glutathione oxide, and 5% glycerol) was performed. The same amounts of His-GSK3β and Dzip1 fragments were mixed in kinase buffer (20 mM Tris-HCl, 10 mM MgCl_2_, 5 mM DTT, 200 μM ATP with 10 μCi γ^32^P ATP [pH 7.5]), and incubated for 30 min at 30°C. Then, loading buffer was added to each sample to stop the reaction. After separation by SDS-PAGE electrophoresis, the gel was exposed to X-ray film at 4°C for 6 h or overnight.

### In Vitro Binding and Semi-Quantifiable GST Pulldown Assays

To examine the Dzip1-mediated dissociation of Rab8^GDP^ from GDI2, GST or GST-GDI2 was incubated with GST affinity binding beads in PBS buffer at 4°C for 1 h. Then, the cell lysate was added to the system for a further 1-h incubation at 4°C. The Rab8-GDI2-coated beads were collected and gently washed three times with PBS, and re-suspended in PBS buffer. The indicated amounts of His-Dzip1 (aa 373–600) or His-Myosin Va (aa 1320–1346) were each added to the system, followed by incubation at 4°C for 2 h. To semi-quantify the role of Dzip1 phosphorylation by GSK3β in regulating the dissociation of Rab8^GDP^ from GDI2, 4, 8, 12, 16, and 20 μg of GST-GDI2 were each preloaded onto the GST affinity binding beads in PBS buffer at 4°C for 1 h, and each group of GDI2-coated beads was further equally divided into four groups (i.e., 1, 2, 3, 4, and 5 μg of GST-GDI2 coated onto beads) for pulldown assays. Each lysate from control, CHIR99021-treated, or S520A- or S520AD-expressing cells was equally divided into five groups and added into the system for a further 1.5-h incubation at 4°C. Finally, the beads were collected and washed four times with IP buffer and analyzed by standard Western blotting.

### Identification of Phospho-Peptide by Mass Spectrometry and Nano-Liquid Chromatography–Tandem Mass Spectrometry

All analyses were performed on an LTQ Orbitrap XL mass spectrometer (Thermo Scientific) at a resolution of 60,000. For nano-liquid chromatography, Eksigent nanoLC 1D plus systems were equipped with 2-mm ReproSil-Pur C18-AQ (Dr. Maisch) trapping columns (packed in house; i.d., 1,150 μm; resin, 5 μm) and 200-mm ReproSil-Pur C18-AQ (Dr. Maisch) analytical columns (packed in house; i.d., 75 μm; resin, 3 μm). The solvents used were 0.5% formic acid-water solution (buffer A) and 0.5% formic acid-acetonitrile solution (buffer B). Trapping was performed at 2 μl/min buffer A for 15 min, and elution was achieved with a gradient of 0%–32% buffer B over 80 min, 32%–50% buffer B over 6 min, and 80% buffer B over 6 min at a flow rate of 300 nl/min. Eluting peptide cations were converted to gas-phase ions by a Nanospray Flex (Thermo Scientific) ion source at 2.0 kV. The mass spectrometer was operated in the data-dependent mode to automatically switch between mass spectrometry (MS) and tandem mass spectrometry (MS/MS). Survey full-scan MS spectra were acquired from m/z 300 to m/z 1,800, and the ten most intense ions with a charge state above 2 and above an intensity threshold of 500 were fragmented in the linear ion trap using a normalized collision energy of 35%. For the orbitrap, the AGC target value was set at 1 × 10^6^, and the maximum fill time for full MS was set at 500 ms. Fragment ion spectra were acquired in the LTQ Orbitrap XL with an AGC target value of 3 × 10^4^ and a maximum fill time of 150 ms. Dynamic exclusion for selected precursor ions was set at 90 s. The lock mass option was enabled for the 462.14658 ion. The raw data were processed using Proteome Discoverer (version 1.4.0.288, Thermo Fisher Scientific). MS2 spectra were searched with the Sequest HT engine against the UniProt mouse complete proteome database (release 2013_06, 50,790 protein sequences). The database was searched with the following parameters: precursor mass tolerance, 20 ppm; MS/MS mass tolerance, 0.6 Da; two missed cleavages for tryptic peptides; dynamic modification oxidation (M), phosphorylation (STY); static modification carbamidomethylation (C). Peptide spectral matches were validated by a targeted decoy database search at 1% false discovery rate. With Proteome Discoverer, peptide identifications were grouped into proteins according to the law of parsimony.

### Statistical Analysis

Statistical analysis was carried out using Microsoft Office Excel 2007. *p-*Values were calculated using the paired *t-*test from the mean values of the indicated data. Significant differences in figures are marked with asterisks (*p < 0.05; **p < 0.01; ***p < 0.001).

## Supporting Information

S1 Data All numerical data used to build histograms and for statistics in this study. (XLSX)

S1 Fig GFP-Dzip1 is concentrated at the basal body and pericentriolar region in NIH 3T3 cells. (A) The Dzip1 antibody Mid1. Mid1, raised by immunizing mice with aa 370–510 of Dzip1, recognized endogenous Dzip1 at ~110 kD both in ciliated NIH 3T3 and non-ciliated HeLa cells. (B) Dzip1 is concentrated at the basal body and the PCM. G0-phase NIH 3T3 cells were immunostained for endogenous Dzip1 with Mid1 and for IFT88. DNA was stained with DAPI. (C) Western blotting analysis of NIH 3T3 cells stably expressing GFP and GFP-Dzip1. MS results demonstrated that the doublet bands recognized by the GFP antibody belonged to Dzip1. (D) GFP-Dzip1 is asymmetrically concentrated in one of the two centrioles. G0-phase NIH 3T3 cells stably expressing GFP-Dzip1 were immunostained for γ-Tubulin (γTub) or AcTub. The DNA was stained with DAPI. Note that the localization of GFP-Dzip1 was similar to that of endogenous Dzip1 shown in Fig 1. (E) Weak centrosome localization of Dzip1 in mitotic cells. Representative immunofluorescence images of cycling NIH 3T3 cells with Dzip1 (antibody Mid2) and PCM1 staining are shown. Note that the centrosomal localization of Dzip1 was weak but still visible in mitosis, but the PCM localization of Dzip1 was undetectable. Also note that Dzip1 was partially co-localized with PCM1 asymmetrically at one of the two spindle poles. Boxes labeled “1” are magnified on right to show centrosomal staining of Dzip1. (F) GFP-Dzip1 is asymmetrically localized to one of the two centrosomes/spindle poles in living mitotic cells. Living mitotic NIH 3T3 cells expressing GFP-Dzip1 were directly visualized under a microscope and images were captured. Note that the centrosomal localization of GFP-Dzip1 persisted during the entirety of mitosis (arrowheads), and that one of the two centrosomes/spindle poles possessed more GFP-Dzip1. Scale bars: 5 μm.
(TIF)

S2 Fig Dzip1 knockdown impairs cilium assembly. (A) Western blotting analysis of NIH 3T3 cells with stable knockdown of Dzip1 (cell lines 1308–3 and 2172–1). Equal amounts of samples were loaded and probed with anti-Dzip1 antibody. GAPDH was probed as a loading control. (B–D) Dzip1 knockdown impairs cilium assembly. The cells without (RNAi control) or with Dzip1 knockdown (1308–3 and 2172–1) were immunostained with Dzip1 and AcTub. The DNA was stained with DAPI. Note that the percentage ciliation ratios and ciliary lengths were both significantly decreased in Dzip1-knockdown cells. Scale bars: 20 μm. (E and F) Full-length GFP-Dzip1 rescues the defect in cilium assembly in Dzip1-knockdown 1308–3 cells. The cells were transfected with GFP or RNAi-resistant full-length GFP-Dzip1 and immunostained with AcTub. The DNA was stained with DAPI. Scale bars: 5 μm. The values in (C), (D), and (F) are mean ± SD; 50 cells were counted in each of three independent experiments. ***p < 0.001.
(TIF)

S3 Fig Dzip1 knockdown does not affect the basal body localization of Rabin8. (A) Dzip1 does not form complexes with IFT88 or γ-Tubulin. G0-phase cells expressing GFP-Dzip1 or GFP were subjected to IP and Western blotting assay with the indicated proteins. White asterisks indicate nonspecific bands. (B) Knockdown of Dzip1 does not affect the localization of Rabin8 to the basal body. Cells without (RNAi Con) and with Dzip1 knockdown (1308–3) were immunostained with Rabin8 and AcTub. Scale bars: 5 μm. (C) Rabin8 does not interact with Dzip1. G0-phase cells expressing GFP-Dzip1 or GFP were subjected to IP and Western blotting assay with Rabin8 and GFP. White asterisks indicate the heavy chain of IgG.
(TIF)

S4 Fig Co-localization of Dzip1 and Rab8, and functional analysis of Dzip1 fragments. (A) Co-localization of Dzip1 and Rab8^GDP^ at the PCM. Basal bodies from G0-phase cells expressing GFP-Rab8^Q67L^ and Flag-Rab8^T22N^ were purified and subjected to 30%–70% sucrose ultracentrifugation. The distributions of the indicated proteins were assessed. The lanes containing PCM1 and Pericentrin indicated that they contained components that belonged to the PCM, while lanes containing Nedd1 and Aurora A were defined as the core of the basal body. Note that most of the Rab8^GDP^ and Rabin8 and a portion of Dzip1 were co-localized at the PCM, but in addition to the PCM localization, Rab8^GTP^ was also localized to the centriole. (B) A scheme of the GFP-Dzip1 fragments. The GFP-tagged Dzip1 fragments were generated and co-expressed with Rab8 or GDI2 in HEK 293T cells. The binding status of Dzip1 fragments with Rab8 and GDI2 are summarized below. (C) The aa 430–600 fragment of Dzip1 is crucial for the binding of Dzip1 and Rab8^GDP^. The proteins immunoprecipitated by GFP-Rab8^T22N^ were probed with the antibody against Myc. (D) His-Myosin Va (aa 1320–1346) does not promote dissociation of the Rab8-GDI2 complex. Increasing amounts of His-Myosin Va (aa 1320–1346) were added to the Rab8-GDI2-coated beads, but this peptide had no effect on decreasing the binding of Rab8 with GDI2. The pulldown proteins and the added peptide were stained with Fast Green. (E) The aa 430–600 fragment of Dzip1 is required for its binding to GDI2. The proteins immunoprecipitated by GFP-Dzip1 fragments were probed with the antibody against Myc. Note that the full-length protein and the 188C fragment interacted strongly with GDI2, whereas the 430C and N600 fragments only weakly bound with GDI2. The quantified band intensities are labeled. (F) Modeling the Rab8^GTP^–Rab^GDP^ gradient from the PCM to the centriole. Rab8, including both active and inactive forms, is localized to the cilium base. Rab8^GTP^ is seen on the cilium membrane and at the basal body (light green); Rab8^GDP^ is accumulated at the PCM but is excluded from the centriole and the cilium membrane (dark green).
(TIF)

S5 Fig Binding of Rab8^GDP^ increases FE^CFP^ of CFP-RBD-YFP. (A) Modeling the CFP-RBD-YFP AB-FRET reporter system (see Materials and Methods). The fusion protein CFP-RBD-YFP produced FRET once stimulated; the FE^CFP^ increased when YFP was bleached (panels a and b). The binding of Rab8^GDP^ changed the distance between CFP and YFP, leading to a change of FE^CFP^ after YFP photo-bleaching caused by the change of FRET between CFP and YFP (panels c and d). (B) CFP-RBD-YFP specifically recognizes Rab8^GDP^. CFP-RBD-YFP immunoprecipitated much more Flag-Rab8^T22N^ than Flag-Rab8^Q67L^ in HEK 293T cells. (C) CFP-RBD-YFP mimics the basal body localization of Rabin8. G0-phase NIH 3T3 cells were immunostained with Rabin8 or CFP-RBD-YFP, and AcTub. (D) Rab8 and Rab8^T22N^ but not Rab8^Q67L^ is co-localized with the CFP-RBD-YFP aggregates in the cytoplasm. Rab8^T22N^ or Rab8^Q67L^ was co-expressed with the CFP-RBD-YFP fusion protein in NIH 3T3 cells. The endogenous or Flag-tagged Rab8 was immunostained with Rab8 or Flag. The DNA was stained with DAPI. (E and F) Binding of CFP-RBD-YFP with Rab8^GDP^ increases the FE^CFP^ after YFP photo-bleaching. Representative images of AB-FRET in one NIH 3T3 cell with three types of CFP-RBD-YFP localization are shown (E). Note that the CFP-RBD-YFP aggregates showed strong FE^CFP^, and the centrosomal CFP-RBD-YFP showed moderate FE^CFP^, whereas the cytoplasmic CFP-RBD-YFP showed no FE^CFP^. The quantitative results of the AB-FRET efficiency of the indicated regions in (E) are shown in (F). Scale bars: 5 μm.
(TIF)

S6 Fig Phosphorylation of Dzip1 by GSK3β promotes the dissociation of Rab8^GDP^ from GDI2. (A) Dzip1 is phosphorylated in G0 phase. Dzip1 in NIH 3T3 cells arrested at G0 phase and prometaphase was analyzed by Western blotting. Note that the up-shifted bands of Dzip1 were evident in G0 phase but abolished by λ-phosphatase (λPPase) treatment. α-Tubulin and histone H3 pS10 were set as loading control and mitosis indicator, respectively. (B) The kinase activity of GSK3β progressively increases from mitosis to G0 phase. NIH 3T3 cells were released from nocodazole-arrested prometaphase into fresh serum-depleted medium, and the indicated proteins were assessed by Western blotting. The expression levels of Cyclin B1 and GAPDH were respectively set as indicators of cell cycle progression and loading control. Note that the expression of GSK3β was steady, but its kinase activity (decrease of pS9) was increased during the mitosis–G0 phase transition. (C) Validation of the specificity of the in vitro phosphorylation assay. The aa 373–600 region of Dzip1 (Mid) was selected as the substrate of GSK3β, and was separately incubated with wild-type His-GSK3β or the K85R mutant in the absence or presence of the GSK3 inhibitors. The bands at ~28 and ~55 kD were the phosphorylated Dzip1 fragments and auto-phosphorylated GSK3β, respectively. Coomassie blue staining of the gel is shown below. Note that phosphorylation of the Dzip1 fragment was abolished either by disruption of the ATP-binding domain of GSK3β (GSK3β^K85R^) or by inhibition of GSK3. (D) Neither GDI2 nor Rab8 is phosphorylated by GSK3β. GST-GDI2, GST, and His-Rab8 were separately incubated with His-GSK3β in the in vitro kinase assay. The bands at ~55 kD (asterisk) were auto-phosphorylated GSK3β (right panel). Coomassie blue staining of the gel is shown on the left panel. (E) The binding of Dzip1 with GDI2 is decreased by GSK3 inhibition. Myc-GDI2 was co-expressed with GFP-Dzip1 in G0-phase HEK 293T cells, followed by IP using an antibody against GFP. (F) Either inhibition of GSK3 or expression of Dzip1^S520A^ leads to increased binding of Rab8 to GDI2. Endogenous Dzip1, Rab8, or GFP-Dzip1 was pulled down by increasing amounts of GST-GDI2 from G0-phase NIH 3T3 or HEK 293T cells. Note that the saturation doses of GST-GDI2 for each of the examined proteins were different. (G) The binding of Rab8 to CFP-RBD-YFP is decreased by GSK3 inhibition. G0-phase HEK 293T cells expressing CFP-RBD-YFP and Flag-Rab8^T22N^ were treated or not treated with the GSK3 inhibitors. The quantified band intensities are respectively labeled in (E) and (G).
(TIF)

S7 Fig Dzip1 phosphorylation by GSK3β is required for cilium assembly in G0-phase cells. (A–C) GSK3 inhibition blocks ciliogenesis in asynchronous cells. The percentage ciliation ratios were calculated in control and CHIR99021-treated NIH 3T3 cells at the indicated time points after serum depletion. Cells treated with BIO or CHIR99021 or not treated were immunostained with γ-Tubulin and AcTub. The DNA was stained with DAPI. The two small boxes are respectively magnified in the top-right boxes. (C) Cilium lengths of the G0-phase cells treated or not treated with the indicated GSK3 inhibitors. The values are mean ± SD; 200 cells were counted in each of three independent experiments. Note that BIO treatment caused abnormal cilium assembly, whereas CHIR99021 treatment totally inhibited cilium assembly. (D) Validation of wild-type, GSK3α^−/−^, and GSK3β^−/−^ MEFs. Equal amounts of cell lysates were loaded and probed with GSK3α or GSK3β. GAPDH was probed as a loading control. (E and F) The kinase activity of GSK3β is required for ciliogenesis. G0-phase GSK3β^−/−^ MEFs were transfected with wild-type GSK3β or mutant S9A, K85R, or R96A. The cells were then immunostained for AcTub, and the DNA was stained with DAPI. The percentage ciliation ratios for (E) are shown in (F). The values in (F) are mean ± SD; 50 pairs of daughter cells were counted in each of three independent experiments. Note that wild type and S9A could fully rescue the cilium assembly failure in G0-phase GSK3β^−/−^ MEFs, whereas R96A partially rescued this failure. (G and H) Expression of Dzip1^S520^ increases the ratio of ciliary Rab8 in GSK3β^−/−^ cells. GSK3β^−/−^ cells were transfected with wild-type GFP-Dzip1 or the mutant S520A or S520D, arrested at G0 phase, and immunostained with Rab8. (I and J) Expression of Dzip1^S520^ rescued the cilium defect in 1308–3 cells. 1308–3 cells were transfected with wild-type GFP-Dzip1 or the mutant S520A or S520D, arrested at G0 phase, and co-immunostained with IFT88 and Rab8. The values in (H) and (J) are mean ± SD; 50 cells were counted in each of two independent experiments. Scale bars: 5 μm. ***p < 0.001.
(TIF)

## Abbreviations

AB-FRET: acceptor-bleaching fluorescence resonance energy transfer
AcTub: acetylated α-tubulin
FE^CFP^: fluorescence emission intensity of CFP
FRET: fluorescence resonance energy transfer
GDF: GDP-dissociating inhibitor protein displacement factor
GDI: GDP-dissociating inhibitor protein
GEF: guanine nucleotide exchange factor
IP: immunoprecipitation
MEF: mouse embryo fibroblast
MS: mass spectrometry
MS/MS: tandem mass spectrometry
PBS: phosphate buffer solution
PCM: pericentriolar matrix
PDB: periciliary diffusion barrier
RNAi: RNA interference
SD: standard deviation

## References

[1] Drummond IA (2012) Cilia functions in development. Curr Opin Cell Biol 24: 24–30. 10.1016/j.ceb.2011.12.007 22226236 PMC3464970

[2] Irigoin F, Badano JL (2011) Keeping the balance between proliferation and differentiation: the primary cilium. Curr Genomics 12: 285–297. 10.2174/138920211795860134 22131874 PMC3131736

[3] Hildebrandt F, Benzing T, Katsanis N (2011) Ciliopathies. N Engl J Med 364: 1533–1543. 10.1056/NEJMra1010172 21506742 PMC3640822

[4] Berbari NF, O’Connor AK, Haycraft CJ, Yoder BK (2009) The primary cilium as a complex signaling center. Curr Biol 19: R526–R535. 10.1016/j.cub.2009.05.025 19602418 PMC2814769

[5] Nachury MV, Seeley ES, Jin H (2010) Trafficking to ciliary membrane: how to get across the periciliary diffusion barrier? Annu Rev Cell Dev Biol 26: 59–87. 10.1146/annurev.cellbio.042308.113337 19575670 PMC2952038

[6] Kim S, Dynlacht BD (2013) Assembling a primary cilium. Curr Opin Cell Biol 25: 506–511. 10.1016/j.ceb.2013.04.011 23747070 PMC3729615

[7] Ishikawa H, Marshall WF (2011) Ciliogenesis: building the cell’s antenna. Nat Rev Mol Cell Biol 12: 222–234 10.1038/nrm3085 21427764

[8] Wang G, Chen Q, Zhang X, Zhang B, Zhuo X, et al (2013) PCM1 recruits Plk1 to the pericentriolar matrix to promote primary cilia disassembly before mitotic entry. J Cell Sci 126: 1355–1365. 10.1242/jcs.114918 23345402

[9] Pugacheva EN, Jablonski SA, Hartman TR, Henske EP, Golemis EA (2007) HEF1-dependent Aurora A activation induces disassembly of the primary cilium. Cell 129: 1351–1363. 17604723 10.1016/j.cell.2007.04.035 PMC2504417

[10] Garcia-Gonzalo FR, Reiter JF (2012) Scoring a backstage pass: mechanisms of ciliogenesis and ciliary access. J Cell Biol 197: 697–709. 10.1083/jcb.201111146 22689651 PMC3373398

[11] Kobayashi T, Dynlacht BD (2011) Regulating the transition from centriole to basal body. J Cell Biol 193: 435–444. 10.1083/jcb.201101005 21536747 PMC3087006

[12] Hsiao Y-C, Tuz K, Ferland RJ (2012) Trafficking in and to the primary cilium. Cilia 1: 4 10.1186/2046-2530-1-4 23351793 PMC3541539

[13] Grigoriev I, Yu KL, Martinez-Sanchez E, Serra-Marques A, Smal I, et al (2011) Rab6, Rab8, and MICAL3 cooperate in controlling docking and fusion of exocytotic carriers. Curr Biol 21: 967–974. 10.1016/j.cub.2011.04.030 21596566

[14] Hattula K, Furuhjelm J, Arffman A, Peranen J (2002) A Rab8-specific GDP/GTP exchange factor is involved in actin remodeling and polarized membrane transport. Mol Biol Cell 13: 3268–3280. 12221131 10.1091/mbc.E02-03-0143 PMC124888

[15] Stenmark H (2009) Rab GTPases as coordinators of vesicle traffic. Nat Rev Mol Cell Biol 10: 513–525. 10.1038/nrm2728 19603039

[16] Pylypenko O, Goud B (2012) Posttranslational modifications of Rab GTPases help their insertion into membranes. Proc Natl Acad Sci U S A 109: 5555–5556. 10.1073/pnas.1202494109 22451945 PMC3326495

[17] Nachury MV, Loktev AV, Zhang Q, Westlake CJ, Peranen J, et al (2007) A core complex of BBS proteins cooperates with the GTPase Rab8 to promote ciliary membrane biogenesis. Cell 129: 1201–1213. 17574030 10.1016/j.cell.2007.03.053

[18] Moore FL, Jaruzelska J, Dorfman DM, Reijo-Pera RA (2004) Identification of a novel gene, DZIP (DAZ-interacting protein), that encodes a protein that interacts with DAZ (deleted in azoospermia) and is expressed in embryonic stem cells and germ cells. Genomics 83: 834–843. 15081113 10.1016/j.ygeno.2003.11.005

[19] Kim HR, Richardson J, van Eeden F, Ingham PW (2010) Gli2a protein localization reveals a role for Iguana/DZIP1 in primary ciliogenesis and a dependence of Hedgehog signal transduction on primary cilia in the zebrafish. BMC Biol 8: 65 10.1186/1741-7007-8-65 20487519 PMC2890509

[20] Tay SY, Yu X, Wong KN, Panse P, Ng CP, et al (2010) The iguana/DZIP1 protein is a novel component of the ciliogenic pathway essential for axonemal biogenesis. Dev Dyn 239: 527–534. 10.1002/dvdy.22199 20014402

[21] Glazer AM, Wilkinson AW, Backer CB, Lapan SW, Gutzman JH, et al (2010) The Zn finger protein Iguana impacts Hedgehog signaling by promoting ciliogenesis. Dev Biol 337: 148–156. 10.1016/j.ydbio.2009.10.025 19852954 PMC2799895

[22] Wang C, Low WC, Liu A, Wang B (2013) Centrosomal protein DZIP1 regulates Hedgehog signaling by promoting cytoplasmic retention of transcription factor GLI3 and affecting ciliogenesis. J Biol Chem 288: 29518–29529. 10.1074/jbc.M113.492066 23955340 PMC3795250

[23] Chen Y, Jiang J (2013) Decoding the phosphorylation code in Hedgehog signal transduction. Cell Res 23: 186–200. 10.1038/cr.2013.10 23337587 PMC3567827

[24] Wakefield JG, Stephens DJ, Tavare JM (2003) A role for glycogen synthase kinase-3 in mitotic spindle dynamics and chromosome alignment. J Cell Sci 116: 637–646. 12538764 10.1242/jcs.00273

[25] Hoeflich KP, Luo J, Rubie EA, Tsao MS, Jin O, et al (2000) Requirement for glycogen synthase kinase-3beta in cell survival and NF-kappaB activation. Nature 406: 86–90. 10894547 10.1038/35017574

[26] Thoma CR, Frew IJ, Hoerner CR, Montani M, Moch H, et al (2007) pVHL and GSK3beta are components of a primary cilium-maintenance signalling network. Nat Cell Biol 9: 588–595. 17450132 10.1038/ncb1579

[27] Mahjoub MR, Xie Z, Stearns T (2010) Cep120 is asymmetrically localized to the daughter centriole and is essential for centriole assembly. J Cell Biol 191: 331–346. 10.1083/jcb.201003009 20956381 PMC2958470

[28] Vieira OV, Gaus K, Verkade P, Fullekrug J, Vaz WL, et al (2006) FAPP2, cilium formation, and compartmentalization of the apical membrane in polarized Madin-Darby canine kidney (MDCK) cells. Proc Natl Acad Sci U S A 103: 18556–18561. 17116893 10.1073/pnas.0608291103 PMC1693701

[29] Graser S, Stierhof YD, Lavoie SB, Gassner OS, Lamla S, et al (2007) Cep164, a novel centriole appendage protein required for primary cilium formation. J Cell Biol 179: 321–330. 17954613 10.1083/jcb.200707181 PMC2064767

[30] Paridaen JT, Wilsch-Brauninger M, Huttner WB (2013) Asymmetric inheritance of centrosome-associated primary cilium membrane directs ciliogenesis after cell division. Cell 155: 333–344. 10.1016/j.cell.2013.08.060 24120134

[31] Anderson CT, Stearns T (2009) Centriole age underlies asynchronous primary cilium growth in mammalian cells. Curr Biol 19: 1498–1502. 10.1016/j.cub.2009.07.034 19682908 PMC3312602

[32] Pazour G, Baker S, Deane J, Cole D, Dickert B, et al (2002) The intraflagellar transport protein, IFT88, is essential for vertebrate photoreceptor assembly and maintenance. J Cell Biol 157: 103–113. 11916979 10.1083/jcb.200107108 PMC2173265

[33] Pazour G, Dickert B, Vucica Y, Seeley E, Rosenbaum J, et al (2000) Chlamydomonas IFT88 and its mouse homologue, polycystic kidney disease gene tg737, are required for assembly of cilia and flagella. J Cell Biol 151: 709–718. 11062270 10.1083/jcb.151.3.709 PMC2185580

[34] Boehlke C, Bashkurov M, Buescher A, Krick T, John AK, et al (2010) Differential role of Rab proteins in ciliary trafficking: Rab23 regulates smoothened levels. J Cell Sci 123: 1460–1467. 10.1242/jcs.058883 20375059

[35] Shisheva A, Chinni S, DeMarco C (1999) General role of GDP dissociation inhibitor 2 in membrane release of Rab proteins: modulations of its functional interactions by in vitro and in vivo structural modifications. Biochemistry 38: 11711–11721. 10512627 10.1021/bi990200r

[36] Roland JT, Lapierre LA, Goldenring JR (2009) Alternative splicing in class V myosins determines association with Rab10. J Biol Chem 284: 1213–1223. 10.1074/jbc.M805957200 19008234 PMC2613619

[37] Doble BW, Woodgett JR (2003) GSK-3: tricks of the trade for a multi-tasking kinase. J Cell Sci 116: 1175–1186. 12615961 10.1242/jcs.00384 PMC3006448

[38] Fumoto K, Hoogenraad CC, Kikuchi A (2006) GSK-3beta-regulated interaction of BICD with dynein is involved in microtubule anchorage at centrosome. EMBO J 25: 5670–5682. 17139249 10.1038/sj.emboj.7601459 PMC1698904

[39] Westlake CJ, Baye LM, Nachury MV, Wright KJ, Ervin KE, et al (2011) Primary cilia membrane assembly is initiated by Rab11 and transport protein particle II (TRAPPII) complex-dependent trafficking of Rabin8 to the centrosome. Proc Natl Acad Sci U S A 108: 2759–2764. 10.1073/pnas.1018823108 21273506 PMC3041065

[40] Frame S, Cohen P, Biondi RM (2001) A common phosphate binding site explains the unique substrate specificity of GSK3 and its inactivation by phosphorylation. Mol Cell 7: 1321–1327. 11430833 10.1016/s1097-2765(01)00253-2

[41] Jin Z, Mei W, Strack S, Jia J, Yang J (2011) The antagonistic action of B56-containing protein phosphatase 2As and casein kinase 2 controls the phosphorylation and Gli turnover function of Daz interacting protein 1. J Biol Chem 286: 36171–36179. 10.1074/jbc.M111.274761 21878643 PMC3196143

[42] Sivars U, Aivazian D, Pfeffer S (2003) Yip3 catalyses the dissociation of endosomal Rab-GDI complexes. Nature 425: 856–859. 14574414 10.1038/nature02057

[43] Dirac-Svejstrup A, Sumizawa T, Pfeffer S (1997) Identification of a GDI displacement factor that releases endosomal Rab GTPases from Rab-GDI. EMBO J 16: 465–472. 9034329 10.1093/emboj/16.3.465 PMC1169650

[44] Machner M, Isberg R (2007) A bifunctional bacterial protein links GDI displacement to Rab1 activation. Science 318: 974–977. 17947549 10.1126/science.1149121

[45] Esters H, Alexandrov K, Iakovenko A, Ivanova T, Thomä N, et al (2001) Vps9, Rabex-5 and DSS4: proteins with weak but distinct nucleotide-exchange activities for Rab proteins. J Mol Biol 310: 141–156. 11419942 10.1006/jmbi.2001.4735

[46] Yu H, Schreiber S (1995) Structure of guanine-nucleotide-exchange factor human Mss4 and identification of its Rab-interacting surface. Nature 376: 788–791. 7651540 10.1038/376788a0

[47] Itzen A, Pylypenko O, Goody R, Alexandrov K, Rak A (2006) Nucleotide exchange via local protein unfolding—structure of Rab8 in complex with MSS4. EMBO J 25: 1445–1455. 16541104 10.1038/sj.emboj.7601044 PMC1440319

[48] Burton J, Burns M, Gatti E, Augustine G, De Camilli P (1994) Specific interactions of Mss4 with members of the Rab GTPase subfamily. EMBO J 13: 5547–5558. 7988552 10.1002/j.1460-2075.1994.tb06892.x PMC395518

[49] Babbey CM, Bacallao RL, Dunn KW (2010) Rab10 associates with primary cilia and the exocyst complex in renal epithelial cells. Am J Physiol Renal Physiol 299: F495–F506. 10.1152/ajprenal.00198.2010 20576682 PMC2944301

[50] Schmidt KN, Kuhns S, Neuner A, Hub B, Zentgraf H, et al (2012) Cep164 mediates vesicular docking to the mother centriole during early steps of ciliogenesis. J Cell Biol 199: 1083–1101. 10.1083/jcb.201202126 23253480 PMC3529528

[51] Kerkela R, Kockeritz L, Macaulay K, Zhou J, Doble B, et al (2008) Deletion of GSK-3beta in mice leads to hypertrophic cardiomyopathy secondary to cardiomyoblast hyperproliferation. J Clin Invest 118: 3609–3618. 10.1172/JCI36245 18830417 PMC2556242

[52] Jackson PK (2011) Do cilia put brakes on the cell cycle? Nat Cell Biol 13: 340–342. 10.1038/ncb0411-340 21460803

[53] Li A, Saito M, Chuang J, Tseng Y, Dedesma C, et al (2011) Ciliary transition zone activation of phosphorylated Tctex-1 controls ciliary resorption, S-phase entry and fate of neural progenitors. Nat Cell Biol 13: 402–411. 10.1038/ncb2218 21394082 PMC4018803

[54] Kim S, Zaghloul N, Bubenshchikova E, Oh E, Rankin S, et al (2011) Nde1-mediated inhibition of ciliogenesis affects cell cycle re-entry. Nat Cell Biol 13: 351–360. 10.1038/ncb2183 21394081 PMC3077088

[55] Hergovich A, Lisztwan J, Thoma CR, Wirbelauer C, Barry RE, et al (2006) Priming-dependent phosphorylation and regulation of the tumor suppressor pVHL by glycogen synthase kinase 3. Mol Cell Biol 26: 5784–5796. 16847331 10.1128/MCB.00232-06 PMC1592755

[56] Lu F, Lan R, Zhang H, Jiang Q, Zhang C (2009) Geminin is partially localized to the centrosome and plays a role in proper centrosome duplication. Biol Cell 101: 273–285. 10.1042/BC20080109 18798731 PMC2782310

