# Supplementary figures and images for: GSK3β-Dzip1-Rab8 Cascade Regulates Ciliogenesis after Mitosis
Source: PLoS Biol. 2015 Apr 10;13(4):e1002129. doi: 10.1371/journal.pbio.1002129 (PMC4393111; doi:10.1371/journal.pbio.1002129)

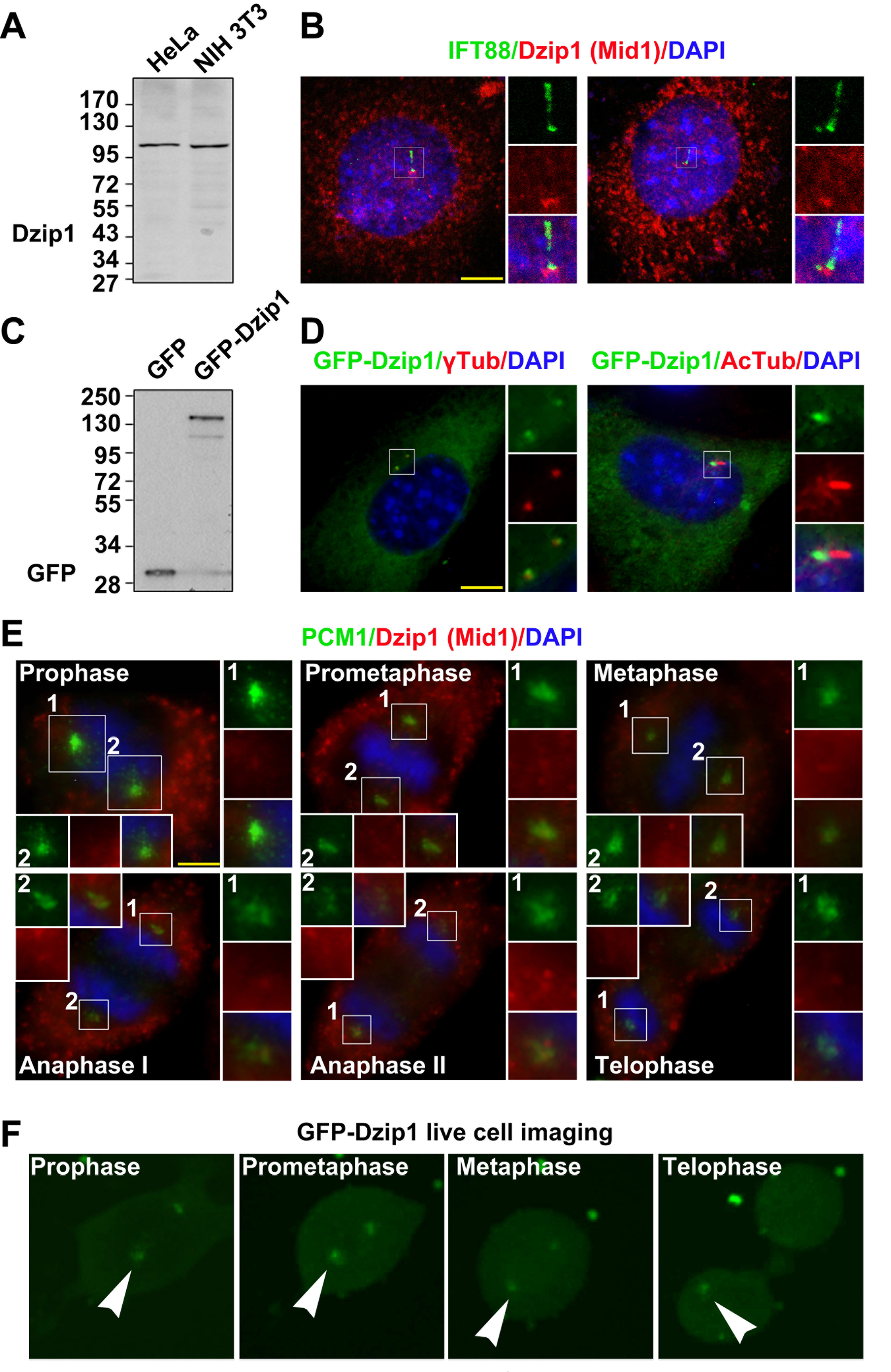

Supplement: S1 Fig — (A) The Dzip1 antibody Mid1. Mid1, raised by immunizing mice with aa 370–510 of Dzip1, recognized endogenous Dzip1 at ~110 kD both in ciliated NIH 3T3 and non-ciliated HeLa cells. (B) Dzip1 is concentrated at the basal body and the PCM. G0-phase NIH 3T3 cells were immunostained for endogenous Dzip1 with Mid1 and for IFT88. DNA was stained with DAPI. (C) Western blotting analysis of NIH 3T3 cells stably expressing GFP and GFP-Dzip1. MS results demonstrated that the doublet bands recognized by the GFP antibody belonged to Dzip1. (D) GFP-Dzip1 is asymmetrically concentrated in one of the two centrioles. G0-phase NIH 3T3 cells stably expressing GFP-Dzip1 were immunostained for γ-Tubulin (γTub) or AcTub. The DNA was stained with DAPI. Note that the localization of GFP-Dzip1 was similar to that of endogenous Dzip1 shown in Fig 1. (E) Weak centrosome localization of Dzip1 in mitotic cells. Representative immunofluorescence images of cycling NIH 3T3 cells with Dzip1 (antibody Mid2) and PCM1 staining are shown. Note that the centrosomal localization of Dzip1 was weak but still visible in mitosis, but the PCM localization of Dzip1 was undetectable. Also note that Dzip1 was partially co-localized with PCM1 asymmetrically at one of the two spindle poles. Boxes labeled “1” are magnified on right to show centrosomal staining of Dzip1. (F) GFP-Dzip1 is asymmetrically localized to one of the two centrosomes/spindle poles in living mitotic cells. Living mitotic NIH 3T3 cells expressing GFP-Dzip1 were directly visualized under a microscope and images were captured. Note that the centrosomal localization of GFP-Dzip1 persisted during the entirety of mitosis (arrowheads), and that one of the two centrosomes/spindle poles possessed more GFP-Dzip1. Scale bars: 5 μm. (TIF) [file pbio.1002129.s002.tif]

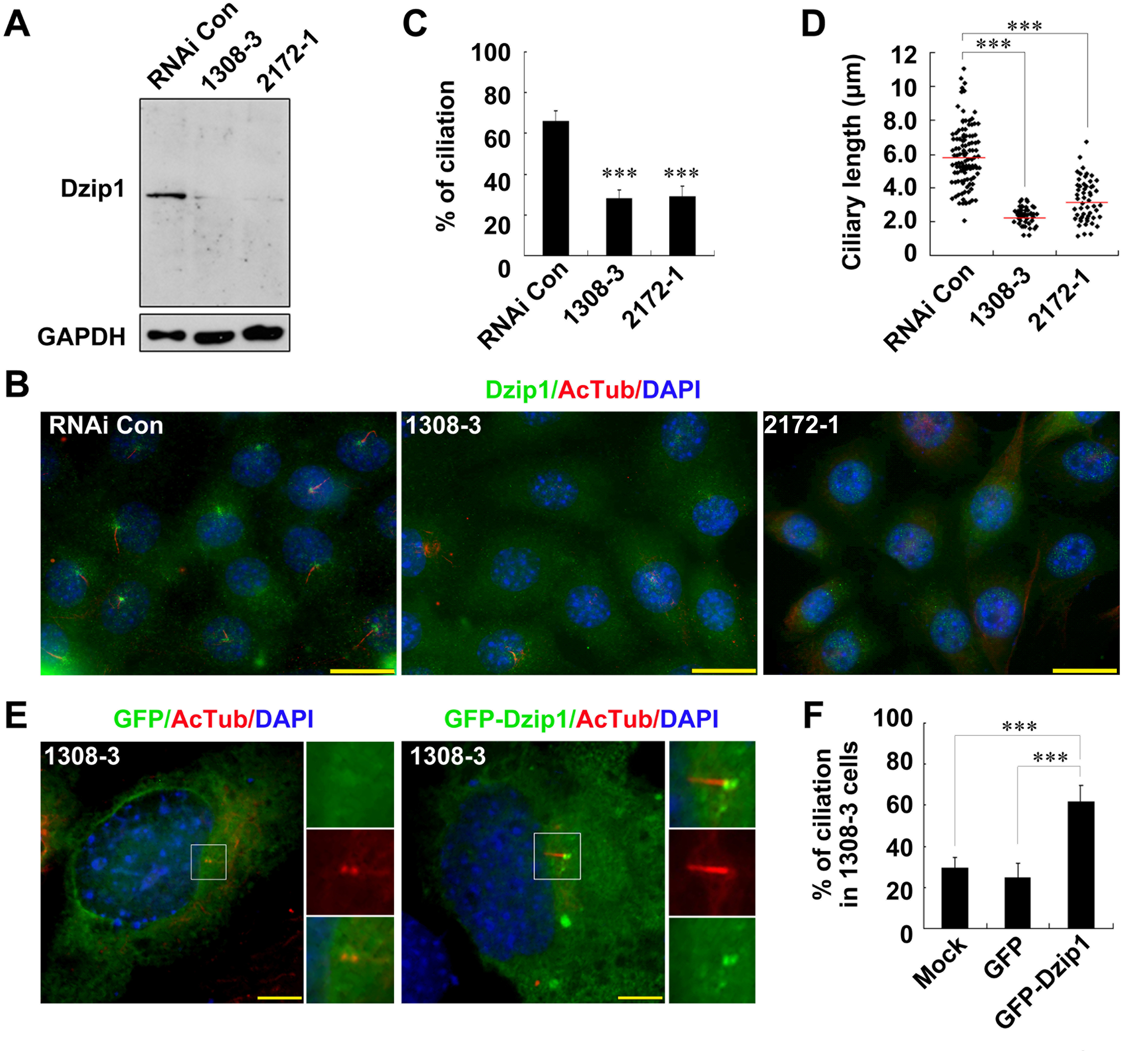

Supplement: S2 Fig — (A) Western blotting analysis of NIH 3T3 cells with stable knockdown of Dzip1 (cell lines 1308–3 and 2172–1). Equal amounts of samples were loaded and probed with anti-Dzip1 antibody. GAPDH was probed as a loading control. (B–D) Dzip1 knockdown impairs cilium assembly. The cells without (RNAi control) or with Dzip1 knockdown (1308–3 and 2172–1) were immunostained with Dzip1 and AcTub. The DNA was stained with DAPI. Note that the percentage ciliation ratios and ciliary lengths were both significantly decreased in Dzip1-knockdown cells. Scale bars: 20 μm. (E and F) Full-length GFP-Dzip1 rescues the defect in cilium assembly in Dzip1-knockdown 1308–3 cells. The cells were transfected with GFP or RNAi-resistant full-length GFP-Dzip1 and immunostained with AcTub. The DNA was stained with DAPI. Scale bars: 5 μm. The values in (C), (D), and (F) are mean ± SD; 50 cells were counted in each of three independent experiments. ***p < 0.001. (TIF) [file pbio.1002129.s003.tif]

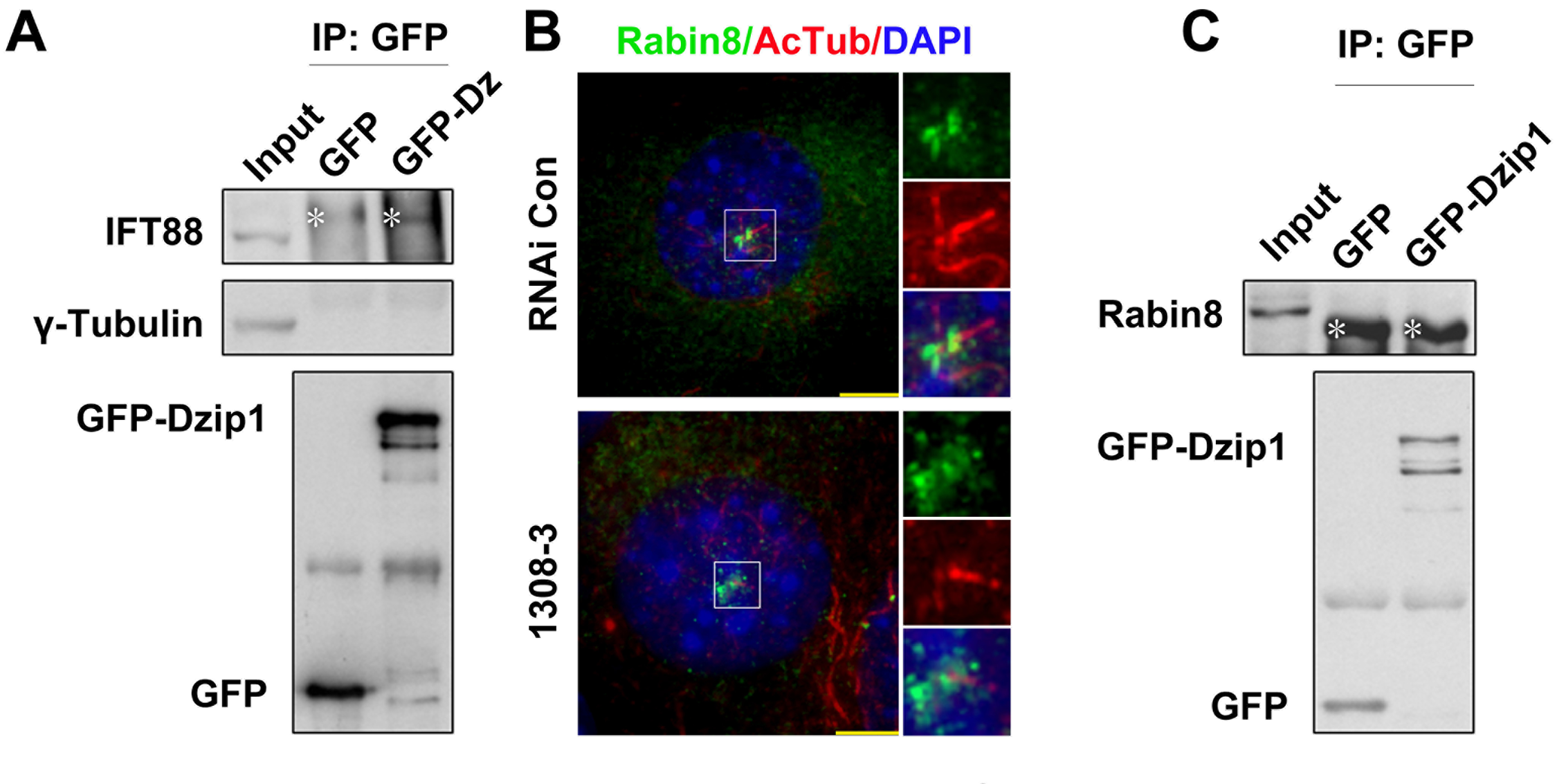

Supplement: S3 Fig — (A) Dzip1 does not form complexes with IFT88 or γ-Tubulin. G0-phase cells expressing GFP-Dzip1 or GFP were subjected to IP and Western blotting assay with the indicated proteins. White asterisks indicate nonspecific bands. (B) Knockdown of Dzip1 does not affect the localization of Rabin8 to the basal body. Cells without (RNAi Con) and with Dzip1 knockdown (1308–3) were immunostained with Rabin8 and AcTub. Scale bars: 5 μm. (C) Rabin8 does not interact with Dzip1. G0-phase cells expressing GFP-Dzip1 or GFP were subjected to IP and Western blotting assay with Rabin8 and GFP. White asterisks indicate the heavy chain of IgG. (TIF) [file pbio.1002129.s004.tif]

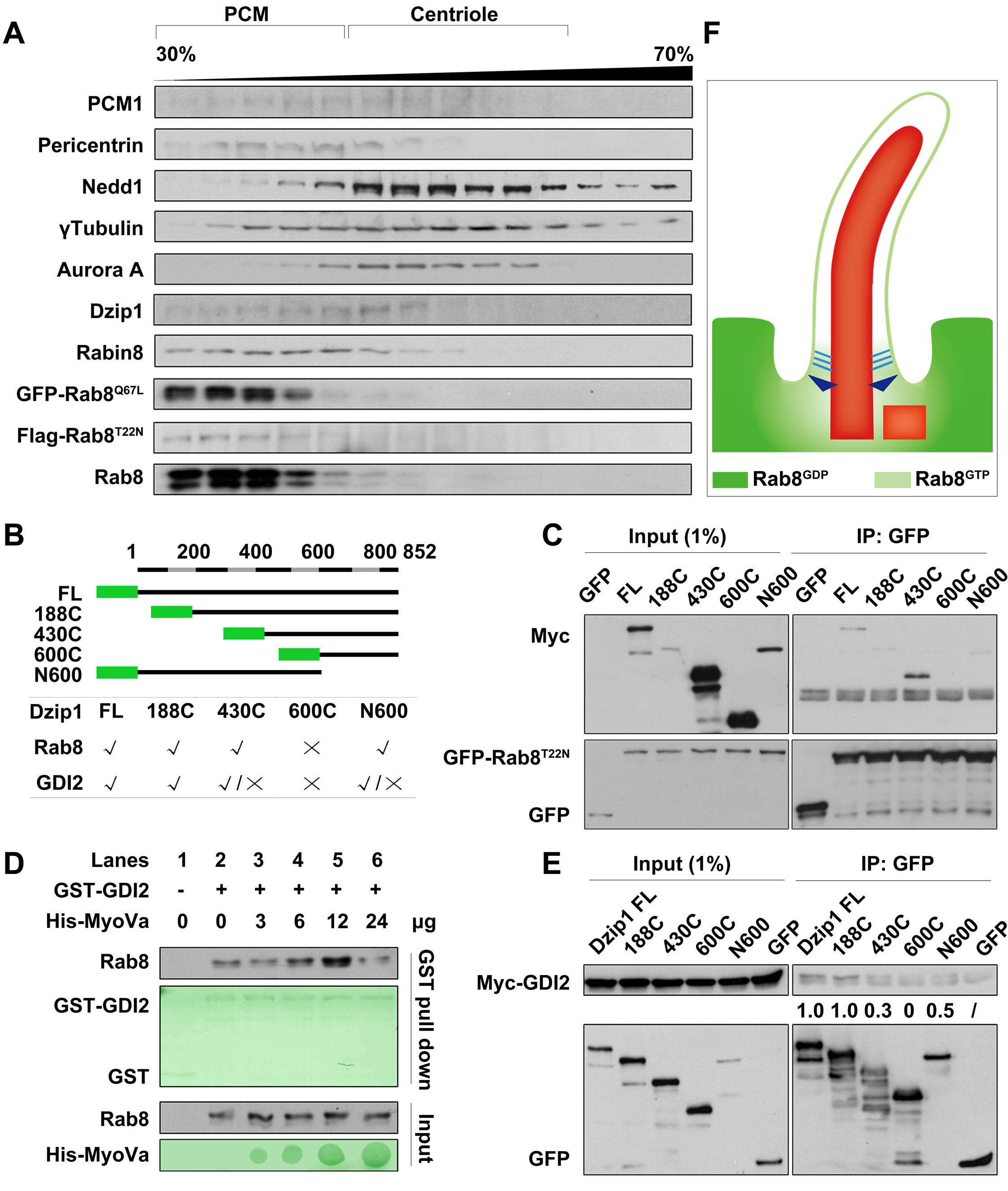

Supplement: S4 Fig — (A) Co-localization of Dzip1 and Rab8GDP at the PCM. Basal bodies from G0-phase cells expressing GFP-Rab8Q67L and Flag-Rab8T22N were purified and subjected to 30%–70% sucrose ultracentrifugation. The distributions of the indicated proteins were assessed. The lanes containing PCM1 and Pericentrin indicated that they contained components that belonged to the PCM, while lanes containing Nedd1 and Aurora A were defined as the core of the basal body. Note that most of the Rab8GDP and Rabin8 and a portion of Dzip1 were co-localized at the PCM, but in addition to the PCM localization, Rab8GTP was also localized to the centriole. (B) A scheme of the GFP-Dzip1 fragments. The GFP-tagged Dzip1 fragments were generated and co-expressed with Rab8 or GDI2 in HEK 293T cells. The binding status of Dzip1 fragments with Rab8 and GDI2 are summarized below. (C) The aa 430–600 fragment of Dzip1 is crucial for the binding of Dzip1 and Rab8GDP. The proteins immunoprecipitated by GFP-Rab8T22N were probed with the antibody against Myc. (D) His-Myosin Va (aa 1320–1346) does not promote dissociation of the Rab8-GDI2 complex. Increasing amounts of His-Myosin Va (aa 1320–1346) were added to the Rab8-GDI2-coated beads, but this peptide had no effect on decreasing the binding of Rab8 with GDI2. The pulldown proteins and the added peptide were stained with Fast Green. (E) The aa 430–600 fragment of Dzip1 is required for its binding to GDI2. The proteins immunoprecipitated by GFP-Dzip1 fragments were probed with the antibody against Myc. Note that the full-length protein and the 188C fragment interacted strongly with GDI2, whereas the 430C and N600 fragments only weakly bound with GDI2. The quantified band intensities are labeled. (F) Modeling the Rab8GTP–RabGDP gradient from the PCM to the centriole. Rab8, including both active and inactive forms, is localized to the cilium base. Rab8GTP is seen on the cilium membrane and at the basal body (light green); Rab8GDP is accumulated at the PCM but is ex [file pbio.1002129.s005.tif]

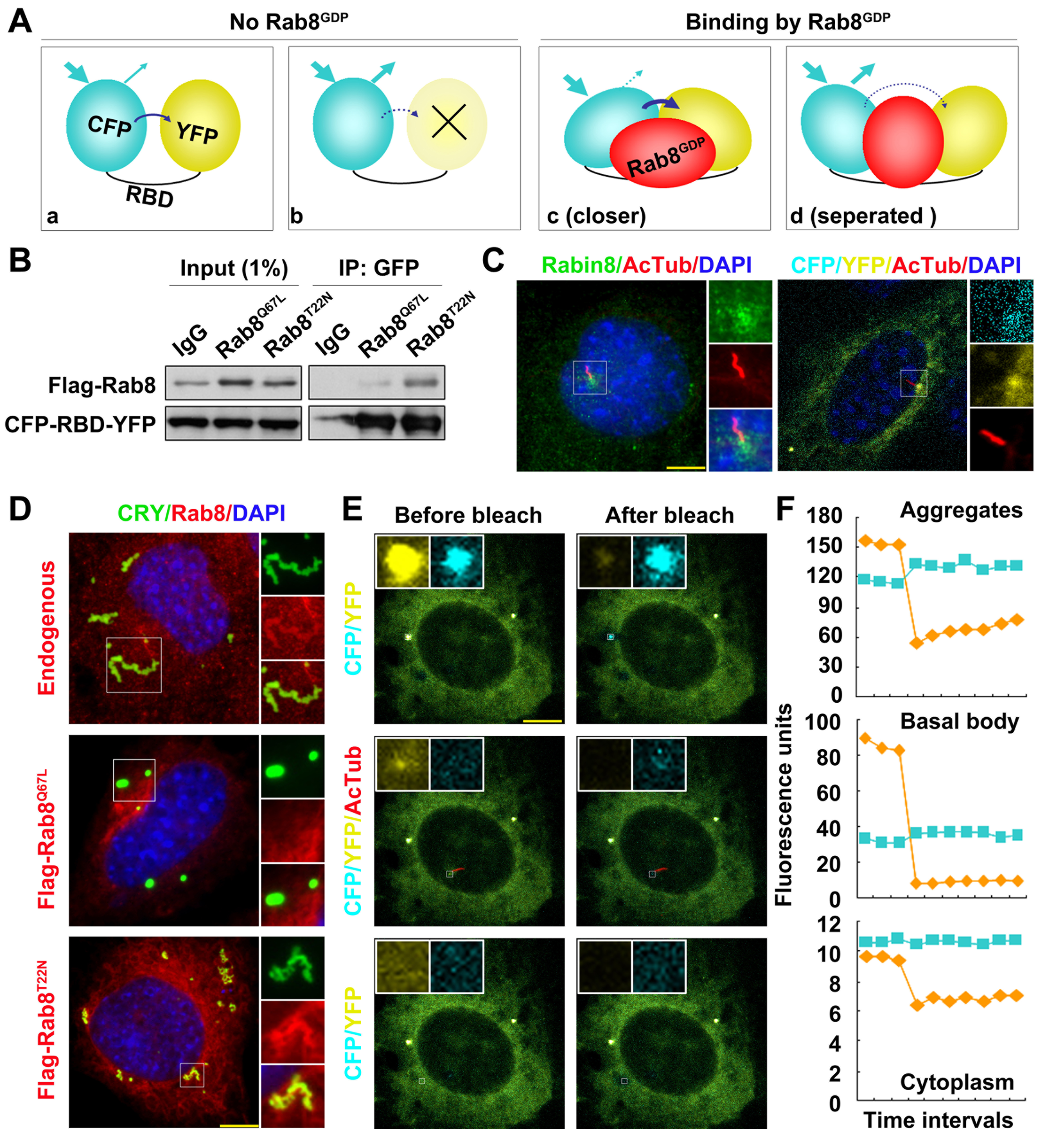

Supplement: S5 Fig — (A) Modeling the CFP-RBD-YFP AB-FRET reporter system (see Materials and Methods). The fusion protein CFP-RBD-YFP produced FRET once stimulated; the FECFP increased when YFP was bleached (panels a and b). The binding of Rab8GDP changed the distance between CFP and YFP, leading to a change of FECFP after YFP photo-bleaching caused by the change of FRET between CFP and YFP (panels c and d). (B) CFP-RBD-YFP specifically recognizes Rab8GDP. CFP-RBD-YFP immunoprecipitated much more Flag-Rab8T22N than Flag-Rab8Q67L in HEK 293T cells. (C) CFP-RBD-YFP mimics the basal body localization of Rabin8. G0-phase NIH 3T3 cells were immunostained with Rabin8 or CFP-RBD-YFP, and AcTub. (D) Rab8 and Rab8T22N but not Rab8Q67L is co-localized with the CFP-RBD-YFP aggregates in the cytoplasm. Rab8T22N or Rab8Q67L was co-expressed with the CFP-RBD-YFP fusion protein in NIH 3T3 cells. The endogenous or Flag-tagged Rab8 was immunostained with Rab8 or Flag. The DNA was stained with DAPI. (E and F) Binding of CFP-RBD-YFP with Rab8GDP increases the FECFP after YFP photo-bleaching. Representative images of AB-FRET in one NIH 3T3 cell with three types of CFP-RBD-YFP localization are shown (E). Note that the CFP-RBD-YFP aggregates showed strong FECFP, and the centrosomal CFP-RBD-YFP showed moderate FECFP, whereas the cytoplasmic CFP-RBD-YFP showed no FECFP. The quantitative results of the AB-FRET efficiency of the indicated regions in (E) are shown in (F). Scale bars: 5 μm. (TIF) [file pbio.1002129.s006.tif]

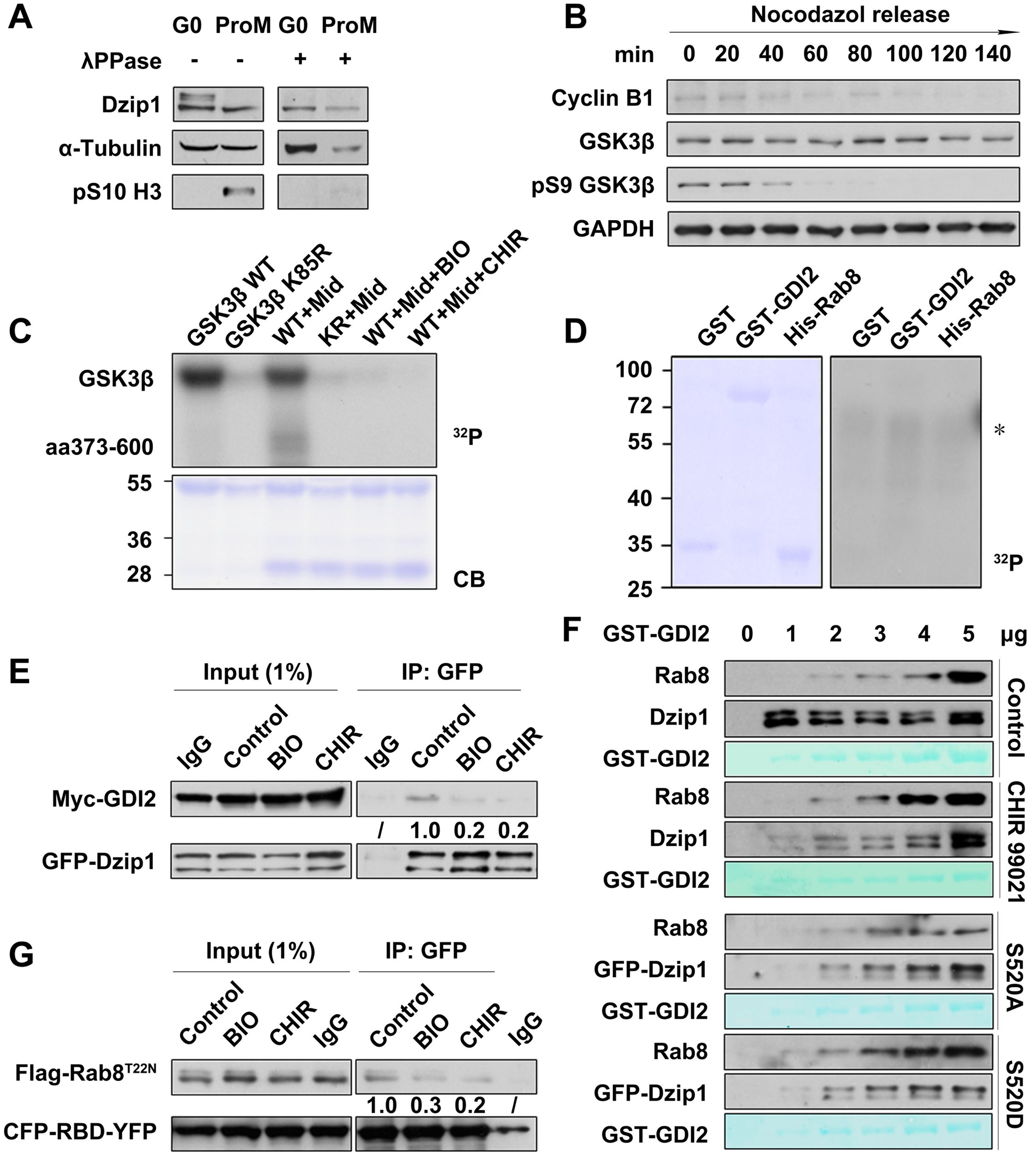

Supplement: S6 Fig — (A) Dzip1 is phosphorylated in G0 phase. Dzip1 in NIH 3T3 cells arrested at G0 phase and prometaphase was analyzed by Western blotting. Note that the up-shifted bands of Dzip1 were evident in G0 phase but abolished by λ-phosphatase (λPPase) treatment. α-Tubulin and histone H3 pS10 were set as loading control and mitosis indicator, respectively. (B) The kinase activity of GSK3β progressively increases from mitosis to G0 phase. NIH 3T3 cells were released from nocodazole-arrested prometaphase into fresh serum-depleted medium, and the indicated proteins were assessed by Western blotting. The expression levels of Cyclin B1 and GAPDH were respectively set as indicators of cell cycle progression and loading control. Note that the expression of GSK3β was steady, but its kinase activity (decrease of pS9) was increased during the mitosis–G0 phase transition. (C) Validation of the specificity of the in vitro phosphorylation assay. The aa 373–600 region of Dzip1 (Mid) was selected as the substrate of GSK3β, and was separately incubated with wild-type His-GSK3β or the K85R mutant in the absence or presence of the GSK3 inhibitors. The bands at ~28 and ~55 kD were the phosphorylated Dzip1 fragments and auto-phosphorylated GSK3β, respectively. Coomassie blue staining of the gel is shown below. Note that phosphorylation of the Dzip1 fragment was abolished either by disruption of the ATP-binding domain of GSK3β (GSK3βK85R) or by inhibition of GSK3. (D) Neither GDI2 nor Rab8 is phosphorylated by GSK3β. GST-GDI2, GST, and His-Rab8 were separately incubated with His-GSK3β in the in vitro kinase assay. The bands at ~55 kD (asterisk) were auto-phosphorylated GSK3β (right panel). Coomassie blue staining of the gel is shown on the left panel. (E) The binding of Dzip1 with GDI2 is decreased by GSK3 inhibition. Myc-GDI2 was co-expressed with GFP-Dzip1 in G0-phase HEK 293T cells, followed by IP using an antibody against GFP. (F) Either inhibition of GSK3 or expression of Dzip1S520A leads to i [file pbio.1002129.s007.tif]

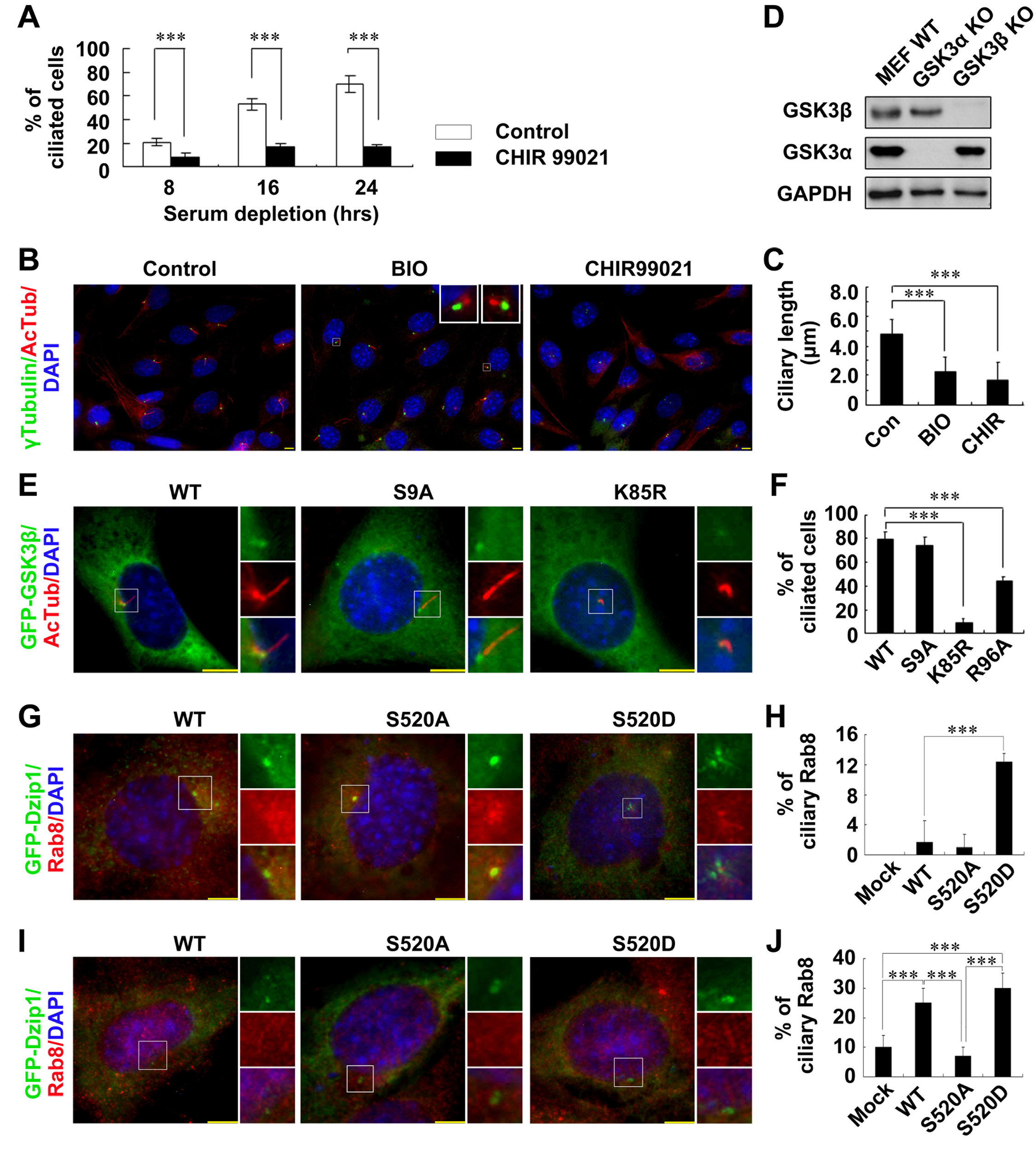

Supplement: S7 Fig — (A–C) GSK3 inhibition blocks ciliogenesis in asynchronous cells. The percentage ciliation ratios were calculated in control and CHIR99021-treated NIH 3T3 cells at the indicated time points after serum depletion. Cells treated with BIO or CHIR99021 or not treated were immunostained with γ-Tubulin and AcTub. The DNA was stained with DAPI. The two small boxes are respectively magnified in the top-right boxes. (C) Cilium lengths of the G0-phase cells treated or not treated with the indicated GSK3 inhibitors. The values are mean ± SD; 200 cells were counted in each of three independent experiments. Note that BIO treatment caused abnormal cilium assembly, whereas CHIR99021 treatment totally inhibited cilium assembly. (D) Validation of wild-type, GSK3α−/−, and GSK3β−/− MEFs. Equal amounts of cell lysates were loaded and probed with GSK3α or GSK3β. GAPDH was probed as a loading control. (E and F) The kinase activity of GSK3β is required for ciliogenesis. G0-phase GSK3β−/− MEFs were transfected with wild-type GSK3β or mutant S9A, K85R, or R96A. The cells were then immunostained for AcTub, and the DNA was stained with DAPI. The percentage ciliation ratios for (E) are shown in (F). The values in (F) are mean ± SD; 50 pairs of daughter cells were counted in each of three independent experiments. Note that wild type and S9A could fully rescue the cilium assembly failure in G0-phase GSK3β−/− MEFs, whereas R96A partially rescued this failure. (G and H) Expression of Dzip1S520 increases the ratio of ciliary Rab8 in GSK3β−/− cells. GSK3β−/− cells were transfected with wild-type GFP-Dzip1 or the mutant S520A or S520D, arrested at G0 phase, and immunostained with Rab8. (I and J) Expression of Dzip1S520 rescued the cilium defect in 1308–3 cells. 1308–3 cells were transfected with wild-type GFP-Dzip1 or the mutant S520A or S520D, arrested at G0 phase, and co-immunostained with IFT88 and Rab8. The values in (H) and (J) are mean ± SD; 50 cells were counted in each of two independent exper [file pbio.1002129.s008.tif]
